# Neurophysiological Effect of Transcutaneous Electrical Spinal Cord Stimulation in Chronic Complete Spinal Cord Injury

**DOI:** 10.1111/aor.15050

**Published:** 2025-06-30

**Authors:** E. L. McNicol, B. Osuagwu, M. Purcell, E. J. McCaughey, C. Lincoln, L. Cope, A. Vučković

**Affiliations:** ^1^ Centre for Rehabilitation School of Engineering, University of Glasgow Glasgow UK; ^2^ Queen Elizabeth National Spinal Injuries Unit Glasgow UK

**Keywords:** motor rehabilitation, neuromodulation, neurophysiology, transcutaneous electrical spinal cord stimulation

## Abstract

**Background:**

Transcutaneous electrical spinal cord stimulation combined with activity‐based therapy (TESCS‐ABT) holds promising potential for motor rehabilitation of spinal cord injury (SCI). However, its effectiveness in individuals with chronic complete SCI remains largely unexplored, despite recent evidence suggesting that many of these individuals exhibit signs of neurological incompleteness. This study was a prospective, single‐arm, open‐label trial that investigated the neurophysiological effects of TESCS‐ABT in chronic complete SCI and assessed whether any changes translate into functional improvements.

**Methods:**

Nine participants with chronic complete SCI were recruited for a two‐phase trial, including 6 weeks of FES‐conditioning and 16 weeks of TESCS‐ABT. Neurophysiological, neurological and functional assessments were conducted at six timepoints throughout the study: baseline; after the FES‐conditioning phase; after 6, 10, and 16 weeks of TESCS‐ABT; and 12 weeks after completion of the intervention.

**Results:**

All participants exhibited increases in synchronized neural signals to muscles in high frequency bands, with limited improvements in corticospinal and spinal excitability. The magnitude and consistency of these neurophysiological changes varied among participants and were influenced by limb dominance. Neurophysiological improvements did not consistently translate into meaningful gains in strength or function.

**Conclusions:**

These findings suggest that the neurophysiological effects of TESCS‐ABT depend on the presence of residual supraspinal connectivity in chronic complete SCI. This SCI population demonstrate a more limited response compared to previous reports in individuals with incomplete SCI. This study provides important insights into the mechanisms and potential limitations of TESCS‐ABT, helping to guide future research toward optimizing the therapy and identifying those most likely to benefit.

**Trial Registration:**

ClinicalTrials.gov identifier: NCT05522920

## Graphical abstract

TESCS‐ABT induced neurophysiological changes in individuals with chronic complete SCI, however these did not consistently translate into functional improvements. These findings highlight the role of residual supraspinal connectivity and inform future research to optimise this neurotechnology for SCI rehabilitation and identify likely responders.

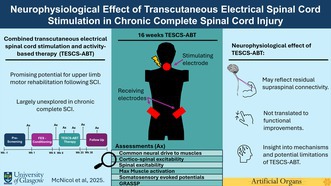

**Abbreviations**
SCI: spinal cord injury
TESCS‐ABT: combined transcutaneous electrical spinal cord stimulation and activity based therapy

## 1 Introduction

The bidirectional disruption of the communication pathway between the brain and muscles caused by spinal cord injury (SCI) can result in the loss of volitional control, sensation and proprioception below the level of injury. Cervical injuries represent the most common and severe type of SCI, accounting for half of all SCI cases [1, 2]. The loss of hand and arm function is one of the most devastating consequences of injury at this level as it severely impacts the person's independence. Consequently, restoring even minimal upper limb function is a top rehabilitation priority for these individuals, as small gains in movement capability can dramatically enhance day‐to‐day independence and overall well‐being [3, 4].

Transcutaneous electrical spinal cord stimulation (TESCS) is a non‐invasive neuromodulatory technique believed to facilitate the transmission of motor signals past the level of injury by enhancing spinal cord excitability [5]. Recent clinical studies have demonstrated the potential of TESCS for promoting functional recovery in individuals with incomplete SCI [6, 7, 8, 9]. While these findings are promising, the mechanisms by which TESCS may support motor recovery are not yet fully understood, and there is insufficient evidence to determine whether similar results can be achieved in individuals with complete SCI.

There is accumulating evidence of residual subclinical transpinal connectivity in some people with spinal cord injuries classified as complete, American Spinal Injury Association Impairment Scale (AIS) A, by standardized clinical sensorimotor examination [10, 11, 12]. Thus, it is thought that sub‐threshold stimulation could promote volitional control via residual descending pathways by enhancing spinal cord excitability, making activation of motor neurons more achievable [13]. This effect is thought to occur through the stimulation of sensory afferents in the dorsal roots, which synapse with interneurons that transmit across multiple different spinal levels, ultimately facilitating motor output. Balaguer et al. challenge the widely accepted notion that spinal cord stimulation facilitates residual supraspinal inputs by increasing motor neuron excitability. Instead, they propose that spinal cord stimulation directly triggers motor neuron action potentials, but only when residual supraspinal drive is present. According to their findings, sub‐threshold sensory inputs generated by spinal cord stimulation are converted into suprathreshold events exclusively in the presence of residual supraspinal input [14]. Despite the differing perspectives on the mechanisms of TESCS, both highlight its potential to improve the efficiency of communication pathways between the brain and muscles [15]. Neurophysiological assessments are critical in this context, as they can reveal changes in neural connectivity and plasticity that may not immediately result in functional improvements but are still significant. These insights could inform and refine rehabilitation therapies, helping to target and harness neuroplastic changes.

The synchronistic neural input to muscles is fundamental to executing controlled and effective motor tasks. Neural drive to muscle refers to the activation signal sent from the pool of innervating motor neurons to initiate muscle contraction, generated by the transmission of synchronized oscillatory inputs into motor neuron outputs. In SCI, this is often disrupted, leading to impaired motor function and diminished capacity for coordinated muscle activation [16, 17, 18, 19]. Coherence analysis is a tool used to assess this neural drive by examining the synchrony of inputs to motor units, providing insights into the organization of neural pathways involved in motor control. Previous studies in individuals with incomplete SCI (iSCI) have demonstrated that improved corticospinal pathways, facilitated by therapies such as locomotor training, can lead to increased beta‐ and gamma‐band inter‐muscular coherence (IMC), reflecting better communication between the cortex and muscles [19, 20]. Locomotor training improves descending motor pathway coordination in iSCI, as evidenced by increased high‐frequency (24–40 Hz) IMC correlating with larger MEP amplitudes [19]. Additionally, preserved corticospinal tract function may enhance motor unit synchronization, reflected in greater beta‐band intramuscular coherence during isometric contractions in individuals with less severe iSCI [20]. Although TESCS is thought to improve the efficiency of the brain‐muscle pathway, the effect of TESCS on common neural drive to muscles has not yet been explored in individuals with SCI. Assessing how TESCS influences common neural drive could reveal crucial insights into how this neuromodulatory technique supports motor control.

Neurophysiological assessments are essential for understanding the underlying neural mechanisms of TESCS and its contribution to motor recovery, particularly in complete SCI, where neurophysiological improvements may not always translate into functional gains. Motor evoked potentials (MEPs) elicited by transcranial magnetic stimulation (TMS) provide valuable insights into the integrity of corticospinal pathways and the ability to transmit motor signals. By measuring both intermuscular coherence (IMC) and MEPs, a comprehensive evaluation of both the functional capacity and structural integrity of the corticospinal pathway can be achieved, as demonstrated in previous studies by Norton and Grossini [19]. Given that supraspinal input is considered critical for TESCS [14], evaluating IMC offers insight into the efficiency of corticospinal drive in relation to pathway integrity. Since TESCS is proposed to enhance corticospinal pathways [15], understanding its impact on both common neural drive and pathway integrity is vital for determining its influence on motor control. Additionally, assessing the integrity of spinal and sensory pathways through spinal motor evoked potentials (sMEPs) and somatosensory evoked potentials (SSEPs), respectively, provides further insight into the neuromodulatory effects of TESCS. Since the median nerve originates from higher spinal segments (C5–C7), changes in SSEP are more likely to be observed in median nerve responses than in ulnar nerve responses (C7–T1) [21]. However, the effect of TESCS on sensorimotor cortical activity remains unclear [22]. While previous studies have demonstrated TESCS‐induced modulation of spinal and corticospinal excitability [15, 23, 24], the exact underlying mechanisms is not yet fully understood, particularly in AIS A SCI. Therefore, combining these neurophysiological assessments offers a more holistic understanding of the neurophysiological effects of TESCS‐ABT, especially in the context of complete SCI.

The aim of this study, as part of a broader investigation, was to explore the neurophysiological effects of TESCS in individuals with chronic complete SCI. Specifically, the aim was to investigate whether TESCS enhances synchronistic neural input to muscles and how this relates to corticospinal excitability. Additionally, spinal excitability, SSEP and muscle activation were assessed to provide a more holistic view of the underlying neurophysiological mechanisms of TESCS. Based on previous findings showing improved communication within corticospinal pathways, we hypothesized that TESCS‐ABT would enhance IMC, which would be associated with increased corticospinal excitability. We anticipated that neurophysiological changes would translate into motor improvements, although the functional significance may be limited due to the high level of SCI in our study population. Nonetheless, evaluating these functional changes provides insight into the potential of TESCS‐ABT to enhance upper limb motor recovery in chronic complete SCI, a population that has been less extensively studied compared to individuals with incomplete SCI. This highlights the importance of investigating neurophysiological changes independently, as improvements may not always translate into observable functional recovery but remain crucial for advancing TESCS‐based rehabilitation approaches. Understanding these neurophysiological changes is essential for uncovering the underlying mechanisms of TESCS that contribute to motor recovery and optimizing its rehabilitative potential.

## 2 Methods

This trial was conducted according to the principles of the Declaration of Helsinki and Good Clinical Practice guidelines. Ethical approval was obtained from the West Midlands—South Birmingham Research Ethics Committee 22/WM/0111. Written informed consent was obtained from participants physically capable of writing and verbal (witnessed) informed consent was obtained from participants unable to write.

### 2.1 Participants

Nine participants with complete cervical (C3–C7) spinal cord injury, AIS A [25], at least 12 months post‐injury were recruited to participate in this trial. Eight participants were enrolled in the study following screening and MRI examination, to identify any syringomyelia prior to enrollment. One participant (P7) was excluded from the study for this reason. Four participants (P2, P4, P5, and P6) completed the entire protocol. Three participants (P3, P8, and P9) withdrew during the first phase of TESCS‐ABT due to personal and health reasons unrelated to this study; and one (P1) withdrew during the last phase of TESCS‐ABT due to personal reasons. Table 1 presents participant demographics, along with their selected muscles of interest and dominant limb.

**TABLE 1:** Participant demographics, muscles of interest and dominant and non‐dominant limbs after injury (determined by ISNCSCI assessment).

Participant	Sex	Time since SCI (years)^a^	NLI	Motor level	ZPP motor	ZPP sensory	Age	Muscle of interest	Dominant limb	Non‐dominant limb
P1	M	21	C2	C4	C7	C4	38	AD and BB	Right	Left
P2	M	5	C2	C4	C4	C4	26	AD and BB	Left	Right
P3	M	1	C1	C5	C5	C4	19	AD and BB	Right	Left
P4	M	12	C4	C5	C7	C7	42	FDS and EDC	Right	Left
P5	M	2	C2	C4	C5	C4	29	AD and BB	Right	Left
P6	M	2	C4	C6	C7	C6	22	FDS and EDC	Left	Right
P7	M	5	C4	C4	N/A	C4	39	N/A	N/A	N/A
P8	M	1	C1	C4	C5	C4	59	AD and BB	Right	Left
P9	M	1	C4	C6	C6	T4	23	FDS and EDC	Right	Left

Abbreviations: AD, anterior deltoid; BB, biceps brachii; EDC, extensor digitorum communis; FDS, flexor digitorum superficialis; NLI, neurological level of injury; ZPP, zone of partial preservation.

^a^ At time of enrollment.

Inclusion criteria specifically included:

1. Willing and able to give informed consent for participation in the study.
2. Aged 18 years or over.
3. Injury level C3–C7, ASIA A.
4. At least 1 year post‐injury.
5. Medically stable, cognitively intact and able to breathe independently.
6. Able to attend all sessions two or three times per week, for 2 h sessions and assessments.

Exclusion criteria specifically included:

1. Any implanted active metallic device including, but not limited to neurostimulators, cochlear implants, pacemakers, implantable defibrillators, or drug delivery pumps.
2. Possible, suspected or confirmed pregnancy and/or lactation.
3. Recent history of fracture, contractures, or pressure sore, DVT, or urinary tract or other infection that may interfere with the intervention and training.
4. History of epilepsy and/or seizures.
5. Severe spasticity which has been unstable over the past 3 months prior to enrollment and not expected to change; taking varying doses of anti‐spasticity medications which could not be tapered to a stable dose and is not expected to change.
6. Botulinum toxin injections within 6 months of participation.
7. Non‐injury related neurological impairment.
8. Clinically significant severe depression in spite of treatment.
9. Has cardiovascular disease.
10. Has autonomic dysreflexia that is severe, ongoing and has required medical treatment within the past 1 month.
11. Skin conditions or allergies that may affect electrode placement.
12. Has had a stem cell treatment or other treatment that could possibly interfere with the outcome of the current study in the past 2 years prior to enrollment.
13. Has been involved in any other study involving stimulation of the spinal cord within 6 months of enrollment.
14. Does not understand English.

### 2.2 Trial Design

The study was designed as a prospective, single‐arm, open‐label trial and was conducted at the Queen Elizabeth National Spinal Injuries Unit, Glasgow.

The trial was divided into two phases. The first phase included functional electrical stimulation (FES) conditioning for up to 1 h, 2–3 times per week (mean ± SD, 16.3 ± 1.1 sessions) for 6 weeks to condition the upper limb muscles. Stimulation was applied using a RehaMove 2 (Hasomed, Germany) FES cycling system at a frequency of 30 Hz, 250 μs pulse width and current amplitude adjusted per channel to achieve the best contraction while minimizing discomfort. Eight channels targeted key muscles of the upper extremities: the elbow flexors (biceps), elbow extensors (triceps), wrist flexors (flexor carpi radialis), and wrist extensors (extensor carpi radialis). Electrode placement was performed on the belly of each targeted muscle, following standard surface electrode placement protocols for optimal contraction. Each session included a 2‐min warm‐up and 2‐min cool‐down of passive cycling, with a target of 30 min of active cycling. During active cycling, FES gradually activated the muscles up to a target speed of 30 rpm or until muscle fatigue was detected. The duration of active cycling, cycling speed, and resistance gradually increased each session, based on the participant's tolerance. This phase was primarily designed to optimize muscle health and function prior to the application of TESCS [26, 27, 28], with the aim of enhancing their ability to perform exercises effectively during the TESCS sessions. Additionally, changes in spinal cord excitability during this period may reveal latent descending influences (voluntary drive) on muscle activity, ultimately enabling these pathways to be trained and strengthened for the subsequent phase.

The second phase involved 16 weeks of TESCS combined with activity‐based therapy (TESCS‐ABT) for up to 2 h, 2–3 times per week (mean ± SD, 40.0 ± 3.7 sessions). Each TESCS‐ABT session consisted of individualized arm and hand exercises, performed with volitional drive, while receiving stimulation. Participants could receive assistance from the therapist to perform activities if required, and a counterweight system and wrist splints were used as necessary. The exercises were selected at the discretion of a SCI specialist occupational therapist or physiotherapist from a list of predefined activities, which can be found in Table S1, based the participant's needs and functional abilities. The difficulty of exercises progressively increased if participants demonstrated improvements, while session duration and stimulation were limited to a maximum of 60 min.

Stimulation was delivered at 30 Hz using a current stimulator (DS8R; Digitimer, UK) with a 10 kHz carrier frequency comprising of trains of 10 biphasic rectangular pulses with a total cycle period of 100 and 40 μs pulse width. The subthreshold current amplitude was adjusted according to the maximum tolerance of the participant (range: 38–100 mA, Figure 2f) [29, 30, 31]. Current intensity was determined by gradually increasing the current until the participant verbally reported it to be too uncomfortable or painful. In all cases, stimulation remained sub‐motor threshold for all participants, as confirmed by the absence of muscle twitches or involuntary movement. No participant tolerated intensities at or above this threshold. A round stimulating cathode electrode (3.2 cm diameter; Axelgaard Manufacturing Co, USA) was attached to the appropriate level of the cervical spine, guided by spinal motor evoked potentials recorded during neurophysiological assessments [7, 31], and two rectangular anode electrodes (8.9 × 5.0 cm; Axelgaard Manufacturing Co, USA) were placed bilaterally over the iliac crests [23]. The placement of the stimulating electrode for each participant was determined by measuring motor‐evoked response of the muscle of interest using single‐pulse spinal stimulation (1 ms pulse width delivered at 1 Hz). Spinal mapping was conducted using a recruitment curve of spinal MEPs relative to the resting motor threshold of the biceps brachii. These spinal maps served as a guide for the SCI specialist occupational therapist or physiotherapist to optimize electrode placement based on the most efficient activation of the targeted muscles during specific activities in TESCS‐ABT sessions. An example spinal map can be found in Figure S1. Although C4–C5 was typically the optimal stimulation site, adjustments were made as needed. The electrode placement for each participant in every TESCS‐ABT session can be found in Figure S2.

Functional, neurological, and neurophysiological assessments, a full list of which is detailed in Table 2, were carried out at six timepoints throughout the study: baseline (base); at the end of the FES‐conditioning phase (FES); after 6 weeks (TESCS_6), 10 weeks (TESCS_10) and 16 weeks (TESCS_16) of TESCS‐ABT intervention; and 12 weeks after completion of the therapy sessions (post_12). The assessments were conducted over three consecutive visits, grouped as follows: cortical motor evoked potentials (cMEP) and EMG dynamometry (neurophys Ax1), sMEP and SSEP (neurophys Ax2), and all functional and clinical assessments (functional and clinical Ax). The order of assessment days was determined by equipment availability and the availability of the SCI specialist occupational therapist or physiotherapist. The EMG during arm cycling was performed during the first therapy session after the three assessment visits and was considered part of the training session.

**TABLE 2:** All assessments performed during the study period.

Neurophysiological assessments	Functional assessments	Clinical assessments
Spinal motor evoked potential	SCIM (spinal cord independence measure) [32]	SCI‐QoL questionnaire [33]
Cortical motor evoked potential	Dynamometry [34]	Modified Ashworth scale (MAS) [35]
Somatosensory evoked potential	Hand function: GRASSP v2 [36, 37]	VAS for pain [38]
Dynamometry with EMG	Daily regular assessment: shoulder shrugs, arm reach‐ups, box and block test [39]	ISNCSCI [25]
EMG during arm cycling		ISCOS–ASA–bladder, bowel, and sexual function [40]

**FIGURE 1:**
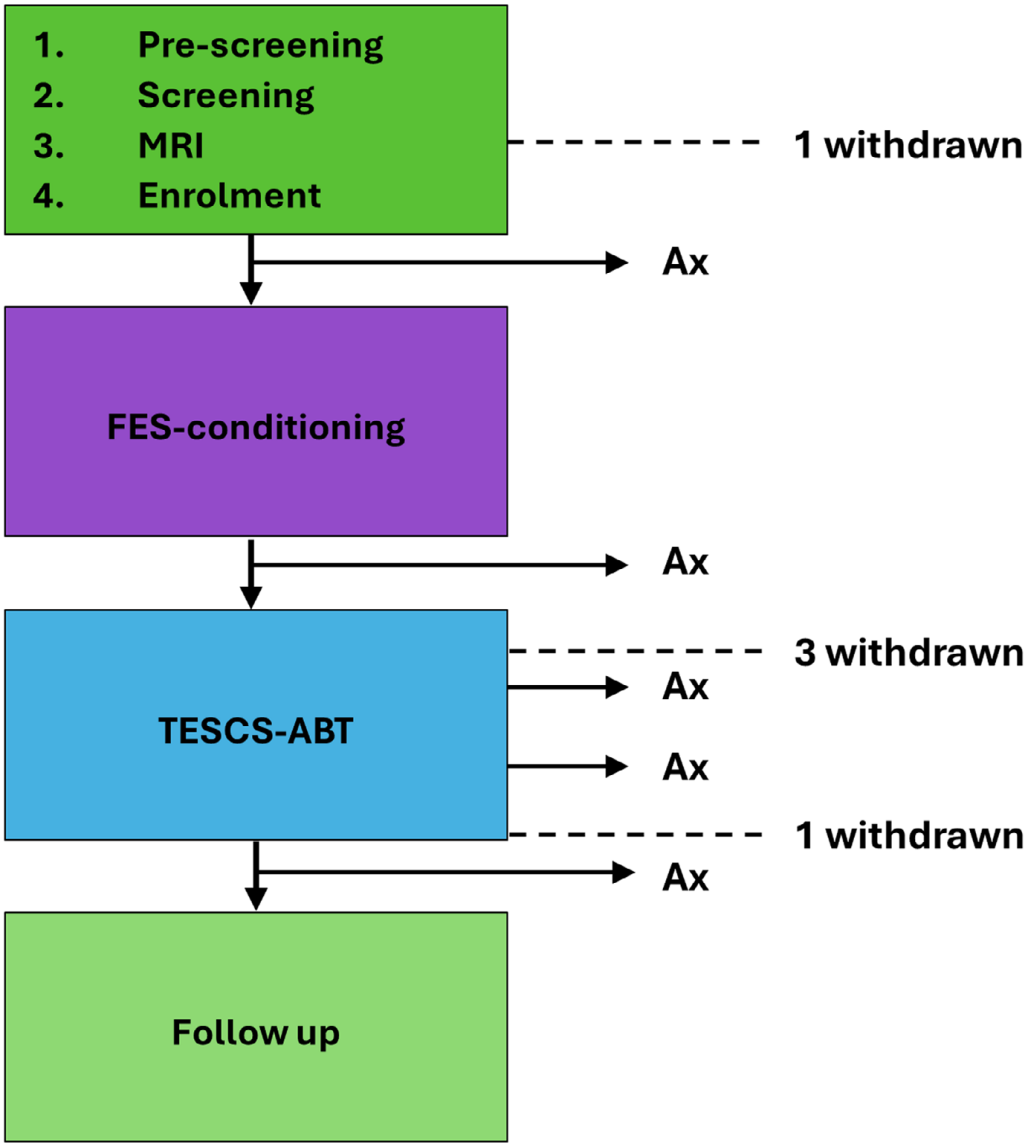
Study order of events. After enrollment, participants engaged in 6 weeks of FES‐conditioning followed by 16 weeks of TESCS‐ABT. Neurophysiological, functional and clinical assessments were carried out at 6 timepoints throughout. The entire study duration was 38 weeks. Ax, assessments. [Color figure can be viewed at wileyonlinelibrary.com]

The study design is illustrated in Figure 1, more details of which can be found on the registered clinical trial page, NCT05522920. This paper focuses on neurophysiological recovery; however, further results from this trial, not discussed in this paper, will be presented elsewhere.

The trial aimed to recruit up to 12 participants, with 8 ultimately enrolled. This sample size reflects the feasibility nature of the trial and was not based on statistical power calculations for outcome detection. However, only 4 participants completed the trial in full and had complete data available for neurophysiological analysis.

### 2.3 Neurophysiological Assessments

#### 2.3.1 EMG Recordings

Bipolar surface EMG electrodes (Biometrics Ltd, UK; surface EMG sensors SX230), with 10 mm diameter and 20 mm inter‐electrode distance, were placed on the muscle belly of a common set of muscle groups relevant to upper limb movements and daily activities. The muscles of interest were selected based on their innervation below the level of injury for each participant, as outlined in Table 1. This approach ensures clinical significance by focusing on muscles where any observed changes are likely to be attributed to the intervention, particularly in participants with preserved function at the injury level. EMG signals were recorded using a DataLINK amplifier (Biometrics Ltd, UK) with a 1000 Hz sampling rate and an internal bandpass filter of 20–460 Hz.

#### 2.3.2 Maximum Voluntary Effort Recordings

Muscle activation during maximum voluntary effort (MVE) attempts using a dynamometer were recorded for biceps brachii (BB), anterior deltoid (AD), flexor digitorum superficialis (FDS), and extensor digitorum communis (EDC) muscles using EMG. These muscles were chosen to account for potential compensatory muscle activation, providing a more comprehensive measure of voluntary effort. Participants were instructed to attempt to squeeze the dynamometer as hard as possible until the assessor requested them to “stop.” Since participants were unable to hold the device independently, the assessor provided support to the dynamometer. This setup offered a tangible reference for the movement, helping participants to engage with the task even in the absence of voluntary grip force. As none of the participants were able to generate measurable grip force, any potential contribution from residual finger muscle activation to the recorded signals was considered negligible. Each MVE trial lasted 3–5 s, with adequate rest intervals to ensure full recovery between attempts. Data collection continued until approximately 2 min of MVE data was obtained.

#### 2.3.3 Motor Evoked Potential Recordings

All MEP data was collected using the EPRecorder (an in‐house MATLAB package, https://github.com/BethelOsuagwu/eprecorder).

##### 2.3.3.1 Cortical MEPs

Transcranial magnetic stimulation (TMS) induced motor evoked potentials (MEPs) were measured from BB, AD, FDS, EDC, first dorsal interosseous (FDI), and abductor digiti minimi (ADM) muscles using EMG. A circular 90 mm Magstim coil (Magstim, UK) was positioned parallel over the vertex (Cz point of the 10–20 system), which was marked with a pen on the scalp to ensure the stimulation site remained constant, with the handle pointing posteriorly along the midline of the head [41, 42]. When recording from the right limb, the coil was oriented to induce an anticlockwise current, preferentially stimulating the left motor cortex. To stimulate the right motor cortex and record from the left limb, the coil was flipped to reverse the current flow. Stimulation was applied in 10% increments from 30% to 50% and then 5% increments from 55% to 100% of maximum stimulator output. Five stimulation pulses were applied at each intensity. If a MEP response was observed in the target muscle, additional pulses were delivered at 120% of RMT to obtain MEP amplitude and construct a stimulus–response curve for that muscle. The FDI was chosen as the target muscle due to its extensive use in TMS research.

While figure‐of‐eight coils provide more focal stimulation, they require precise hotspot identification, which can be time consuming and prone to inadvertent displacement. Whereas the circular coil positioned at the vertex allows for reliable stimulation of large and superficial motor areas, including those responsible for hand and forearm muscles. This setup allows consistent and reproducible stimulation, as the coil can be easily realigned with standard 10–20 EEG system coordinates across sessions. Prior studies have demonstrated comparable efficacy between circular and figure‐of‐eight coils for eliciting MEPs from upper‐limb muscles [41, 43, 44]. Given these considerations, vertex stimulation with a circular coil offers a practical, reliable, and reproducible method for assessing cortical excitability in upper‐limb muscles, particularly in a clinical research settings, where reproducibility and ease of use are critical.

##### 2.3.3.2 Spinal MEPs

Spinal MEPs were recorded bilaterally from BB, FDS and EDC muscles using EMG. Electrical stimulation was delivered using the DS7 Digitimer stimulator (Digitimer, UK), with rectangular single pulses of 1 ms duration at a frequency of 1 Hz or lower. The stimulation intensity was determined relative to the resting motor threshold (RMT) of the target muscle. To establish the RMT, the stimulation electrode was initially placed over the C3–C4 region, targetting the biceps brachii with the lowest RMT. If no detectable response was observed in the biceps brachii of either limb, the target muscle was changed to the FDS, which was then used as the target muscle for all subsequent sMEP assessments. The biceps brachii was selected due to its functional relevance in elbow flexion and forearm supination, both essential for upper limb function and daily activities, as well as its high likelihood of eliciting a response in all participants. RMT was defined as the lowest stimulus intensity that elicited a peak‐to‐peak sMEP amplitude of at least 50 μV in at least 5 out of 10 consecutive trials. After determining the RMT, electrical stimulation was applied sequentially across spinal levels: C3–C4, C4–C5, C5–C6, C6–C7, and C7–T1. Stimuli were delivered in 10% increments across a range of 20%–160% of the BB RMT, with three stimulations performed at each intensity level.

Evoked potentials from supra‐maximal peripheral nerve stimulation were used to normalize spinal MEPs. Single pulse stimulation with a 1 ms pulse width was delivered using the DS7 Digitimer stimulator (Digitimer, UK) to the left and right brachial plexus at Erb's point, the median nerve at the elbow, and the radial nerve at the elbow. Stimulation was applied via two round electrodes (3.2 cm diameter) placed at the appropriate locations. The stimulation amplitude was gradually increased until the evoked response of the indicated muscles (BB for the brachial plexus, FDS for the median nerve, and EDC for the radial nerve) stopped increasing. Once the response plateaued, three stimulations were applied, and the evoked potential was recorded using EMG.

#### 2.3.4 Somatosensory Evoked Potentials

SSEP was measured for the left and right median and ulnar nerves, as these two nerves share innervation of the wrist and fingers. All four nerves were stimulated separately, one at a time. Single pulse electrical stimulation was delivered using the Digitimer DS7 stimulator (Digitimer, UK) with rectangular biphasic pulse of 200 μs. Stimulating electrodes (3.2 cm; Axelgaard Manufacturing Co, USA) were attached on the surface of the skin above the corresponding nerves at the wrist. Stimulation intensity was increased until twitches were observed in the thumb for the median nerve and little finger for the ulnar nerve. For each nerve, a total of 300 stimuli was delivered at the intensity at a frequency of 3 Hz.

EEG was recorded at 4800 Hz with a g.USBamp biosignal amplifer (g.tec, Austria) during stimulation. SSEP of the right hand median and ulnar nerve was measured at CP3 and of the left hand nerves at CP4 (according to the international 10–20 electrode positioning system) using g.LADYbird active EEG electrodes. Additional electrodes were placed over the left and right Erbs points and C6 spinal cord level. The ground and reference were placed at FpZ and Fz respectively. EEG was internally bandpass filtered at 0.5–500 Hz and notch filtered at 50 Hz.

### 2.4 GRASSP Assessment

The GRASSP v2 (graded redefined assessment of strength, sensibility and prehension) test was carried out by trained clinicians, with extensive experience in rehabilitation for people with SCI, at each assessment timepoint to evaluate hand function [36, 37].

The minimal detectable difference (MDD) represents the smallest real change beyond measurement error. For GRASSP V2, the MDD values per side have been determined as: 4 points or more for sensation, 5 points or more for strength, 4 points or more for prehension ability, and 3 points or more for prehension performance [37]. The minimal clinically important difference (MCID) is another key measure for assessing meaningful clinical change. It is defined as the smallest change that individuals receiving treatment consider significant. In chronic complete SCI, the MCID for GRASSP measures has been reported as 2–3 points [45]. While the MDD serves as an important benchmark for detecting statistically significant changes, the MCID represents a more patient‐centered measure of meaningful change, reflecting what patients themselves consider a clinically relevant improvement.

Although it is crucial to understand the perceived benefit of change from the perspective of people living with SCI, estimating the MCID can be challenging due to the variability in individual responses and the difficulty in determining what constitutes a meaningful change for each person. In some cases, the MCID may be smaller than the MDD, particularly when even small changes can have substantial clinical significance, as is often the case in individuals with cervical SCI. For these patients, even small improvements in upper limb function can significantly improve their quality of life [3, 4].

Therefore, while a valid MCID should generally exceed the MDD to ensure that the observed change is both measurable and clinically relevant [46], it is important to recognize that the specific context of the patient population under study may justify using an MCID that is lower than the MDD, especially when small changes can have a significant impact on the person's quality of life.

### 2.5 Data Analysis

#### 2.5.1 Adherence

Adherence to the study protocol was assessed by considering the attendance during each therapy block and the timing of outcome measure assessments. Therapy blocks were considered as the sessions between outcome assessments: 6 weeks of FES‐conditioning sessions (FES); 6 weeks TESCS‐ABT sessions (TESCS‐ABT 1); 4 weeks TESCS‐ABT sessions (TESCS‐ABT 2); and 6 weeks TESCS‐ABT sessions (TESCS‐ABT 3).

Adherence to a therapy block (*a*) was calculated using Equation (1), where *x* represents the number of sessions attended and *s* represents the total number of sessions in the therapy block as per the study protocol.

(1) $$a=\frac{x}{s};a\in \left[0,1\right]$$

Adherence to the timing of the assessment visits (*b*) for outcome measures (grouped into neurophys Ax1, neurophys Ax2 and functional and clinical Ax) was calculated using Equation (2), where *w* denotes the time delay (in weeks) between the original schedule of assessments as per the study protocol and the actual schedule.

(2) $$b=\frac{1}{\left\lfloor w\right\rfloor +1};b\in \left[0,1\right]$$

The study phase adherence (*c*), accounts for the knock on effect between a therapy block and the corresponding assessment timepoint. It is calculated using Equation (3), assuming equal weighting for simplicity. This is valid for post‐baseline and pre follow‐up. By definition, at baseline c≔1 and at follow‐up c≔1/b.

(3) $$c=\frac{a}{2}+\frac{b}{2}=\frac{x}{2s}+\frac{1}{2\left\lfloor w\right\rfloor +2};c\in \left[0,1\right]$$

The overall study protocol adherence (*C*) was calculated as the average phase adherence across all study phases, assuming equal importance of each phase, Equation (4), where *N* is the number of study phases including baseline and follow‐up.

(4) $$C=\frac{1}{N}\sum_{k=1}^{N}c_{k}$$

The relative adherence was also calculated to compare successive study phases without accounting for knock‐on effects. To calculate relative adherence, the adherence to the timing of the assessment visits (denoted by Equation 5) was modified as detailed in Equation (5), where *k* and *j* denote study phases corresponding to the assessments.

(5) $${\bar{b}}_{k}=\frac{1}{\left\lfloor w_{k}-w_{j}\right\rfloor +1}$$

Therefore, the relative adherence at study phase *k* relative to study phase *j* is given by Equation (6), where $\left\{{\bar{C}}_{k};k=1,2,3,\ldots ,N\right\}$ represents a series of successive relative adherence values. By definition, at baseline $\bar{C}≔1$. and at follow‐up, $\bar{C}≔1/\bar{b}$.

(6) $${\bar{C}}_{k}=\frac{a_{k}}{2}+\frac{{\bar{b}}_{k}}{2}=\frac{x}{2s_{k}}+\frac{1}{\left\lfloor w_{k}-w_{j}\right\rfloor +1}$$

#### 2.5.2 Maximum Voluntary Effort

EMG data during MVE trials was filtered offline for powerline noise using a notch filter (48–52 Hz) based on Discrete Fourier Transform (DFT). Data was subsequently visually epoched per contraction period and concatenated prior to calculating inter‐muscular coherence (IMC) and MVE amplitude of muscles of interest (Table 1).

##### 2.5.2.1 Inter‐Muscular Coherence

Inter‐muscular coherence (IMC) analysis offers a way to assess common neural drive by examining the synchronized neural input to motor units of two separate muscles. Shifts in IMC within physiologically relevant frequency bands (e.g., beta and gamma) can reveal insights into the efficiency of spinal pathways involved in voluntary motor control. IMC between muscle pairs of interest (Table 1) was calculated using MATLAB NeuroSpec version 2.0 scripts (www.neurospec.org), applying Equation (7) for coherence estimates. Where the coherence value, *C*(*ω*), at each frequency, *ω*, is given by the cross spectrum of the two EMG signals (*x* and *y*), *S*_*xy*_, normalized by the power spectra of signal *x*, *S*_*xx*_ and signal *y*, *S*_*yy*_.

(7) $$C\left(\omega \right)=\frac{|S_{\mathrm{xy}}{\left(\omega \right)}^{2}|}{S_{\mathrm{xx}}\left(\omega \right)S_{\mathrm{yy}}\left(\omega \right)}$$

For each participant, significant IMC estimates exceeding the 95% confidence limit were summed across frequency bands of interest: beta (15–30 Hz), low gamma (30–48 Hz), and high gamma (52–98 Hz). These bands are neurophysiologically relevant, covering ranges commonly analyzed in coherence studies, allowing for comparisons with existing research. The alpha band, though relevant due to its spinal origin, was excluded due to the internal EMG bandpass filter cutoff at 20 Hz. Only coherence estimates above the 95% confidence threshold were included, as values below this threshold suggest no significant linear association between signals, where zero coherence is considered plausible at that frequency [47]. The confidence limits (CI) were calculated using Equation (8), where *L* represents the number of non‐overlapping segments in the spectral estimation [47]. IMC was analyzed as the sum of coherence (IMC_area_) across each frequency band of interest, which has been shown to be more physiologically meaningful than focusing solely on coherence peaks [48, 49]. Finally, data normalization over the frequency bands was conducted as detailed in Equations ((9), (10), (11)), where *A*_coh_ (*β*) is the normalized sum of IMC across the beta band, 15–30 Hz, *A*_coh_(*γ*_low_) is the normalized sum of IMC across the low gamma band, 30–48 Hz, and *A*_coh_(*γ*_high_) is the normalized sum of IMC across the high gamma band, 52–98 Hz.

(8) $$\mathrm{CI}=1-{\left(0.05\right)}^{\frac{1}{L-1}}$$

(9) $$A_{\mathrm{coh}}\left(\beta \right)=\frac{\sum_{f=15}^{30}\mathrm{IMC}}{15}$$

(10) $$A_{\mathrm{coh}}\left(\gamma_{\mathrm{low}}\right)=\frac{\sum_{f=30}^{48}\mathrm{IMC}}{18}$$

(11) $$A_{\mathrm{coh}}\left(\gamma_{\text{high}}\right)=\frac{\sum_{f=52}^{98}\mathrm{IMC}}{46}$$

##### 2.5.2.2 MVErms

To measure the magnitude of muscle activity, the pre‐processed EMG data from MVE trials was full wave rectified and the root mean square (RMS) of the signal was calculated. Baseline noise was subsequently removed by subtracting the average RMS of a 1 s resting signal before calculating the maximum RMS for each muscle.

#### 2.5.3 Motor Evoked Potentials

The EPRecorder was used to pre‐process the MEP data, allowing recordings to be easily visualized, cleaned and epoched. Each dataset was epoched between 100 ms pre‐stimulation to 100 ms post‐stimulation. Trials were then manually reviewed and excluded if they displayed significant noise or excessive background activity, assessed by comparing the statistical properties of the pre‐stimulus and post‐stimulus periods. MEP responses were defined as those with a peak‐to‐peak amplitude of at least 50 μV and were used to determine the resting motor threshold (RMT). The RMT for each muscle was defined as the smallest intensity that elicited a response greater than 50 μV peak to peak amplitude in at 50% of trial stimuli. To ensure accurate and consistent MEP amplitude calculations, RMT was reassessed at each time point throughout the study.

Peak‐to‐peak MEP amplitude was calculated at 110% RMT and normalized with the root mean square of a 3 s maximum voluntary effort attempt and supra‐maximal peripheral nerve stimulation of the median nerve at the elbow for cMEPs and sMEPs, respectively. This approach ensured that stimulation remained suprathreshold relative to individual excitability at each session, thereby minimizing variability from day‐to‐day threshold changes and maintaining consistent physiological relevance across time points. While using a fixed stimulation intensity would control for absolute output, it could result in sub‐ or suprathreshold stimulation due to shifts in RMT, potentially confounding the interpretation of MEP amplitude changes. To confirm that observed changes in MEP amplitude were not driven by stimulation intensity, Spearman's rank correlation analyses were conducted between RMT and cortical MEP amplitudes, as well as between RMT and sMEP amplitudes, across all sessions and participants.

Since no cortical MEP response was observed in the FDI for any participant throughout the study, analysis was based on the available data. Consequently, cortical MEPs from the muscles of interest, detailed in Table 1, were analyzed for each participant.

For sMEP analysis, only the FDS muscle was used, as supra‐maximal peripheral nerve stimulation data for FDS normalization were available for all sessions and participants. In contrast, corresponding data for the BB and EDC muscles were not consistently available.

The correlation between high gamma‐band IMCarea and cMEP amplitude was considered by calculating Spearman's correlation coefficient. Given the small sample size, data from all available assessment sessions across participants were combined to increase statistical power for the correlation analysis.

## 3 Results

Four participants completed the entire study protocol.

An overview of participant results from neurophysiological and GRASSP assessments is detailed in Table 3. The table presents whether there was a presence of high gamma‐band IMC, RMT, and MEP response from TMS, MVErms above 0, MEP response from spinal stimulation at any spinal level, SSEP response, and GRASSP scores above 0 for each participant in at least one muscle of interest for the left and right limbs at baseline. It also indicates whether these outcomes improved after 16 weeks of TESCS‐ABT compared to after the FES conditioning period, and if any improvements were sustained at the 12‐week follow‐up in at least one muscle of interest for the left and right limb.

**TABLE 3:** Overview of participant results.

	Participant 1						Participant 2						Participant 3						Participant 4						Participant 5						Participant 6						Participant 8						Participant 9					
	Base		Post–pre		Post_12		Base		Post–pre		Post_12		Base		Post–pre		Post_12		Base		Post–pre		Post_12		Base		Post–pre		Post_12		Base		Post–pre		Post_12		Base		Post–pre		Post_12		Base		Post–pre		Post_12	
	L	R	L	R	L	R	L	R	L	R	L	R	L	R	L	R	L	R	L	R	L	R	L	R	L	R	L	R	L	R	L	R	L	R	L	R	L	R	L	R	L	R	L	R	L	R	L	R
Neurophys Ax																																																
IMC		X		X					X				X	X					X	X	X	X	X	X	X	X	X	X	X	X		X					X	X		X			X	X				
cMEP RMT	X	X		X					X		X		X	X					X	X	X	X	X	X	X	X					X	X					X	X					X	X				
cMEP amp	X	X		X					X		X		X	X					X	X	X				X	X					X	X		X		X	X	X					X	X				
MVErms			X								X		X	X					X	X	X	X		X	X	X	X	X	X	X	X	X			X	X	X	X					X	X				
sMEP amp	X	X	X	X					X				X	X					X	X			X	X	X	X		X			X	X	X	X			X	X		X			X					
SSEP																																																
GRASSP Ax																																																
Strength		X	X	X				X	X		X		X	X					X	X					X	X		X		X	X	X					X	X	X				X	X				
Sensation							X		X											X		X									X	X		X		X							X	X				
Prehension ab																				X											X	X	X		X								X	X				
Prehension perf																				X		X		X							X	X											X	X				

*Note:* X denotes the presence of response at baseline (base columns), improvement after intervention compared to before (post–pre columns) and sustained improvement at follow up (post_12 columns) in at least one muscle of interest. Shaded gray boxes denote no response or improvement and blank boxes represent unavailable data.

Abbreviations: GRASSP Ax, GRASSP assessments; L, left limb; Neurophys Ax, neurophysiological assessments; prehension ab, prehension ability; prehension perf, prehension performance; R, right limb.

**FIGURE 2:**
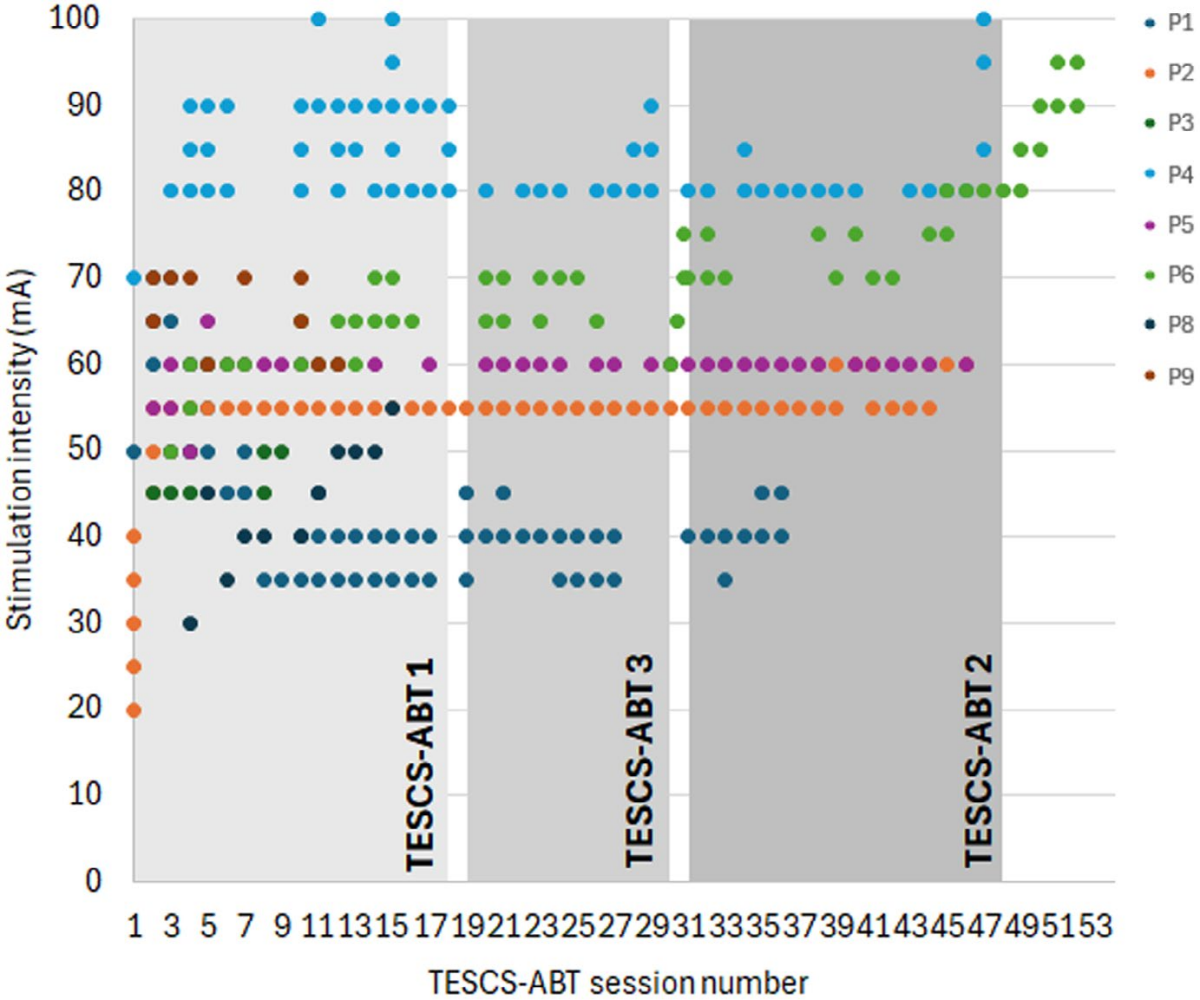
Participant stimulation intensities for each session during the TESCS‐ABT study phases. Values represent peak current amplitudes; the use of a high‐frequency carrier and short pulse width may necessitate higher amplitudes compared to conventional continuous biphasic stimulation protocols. [Color figure can be viewed at wileyonlinelibrary.com]

Participant stimulation intensity used for each session throughout the different TESCS‐ABT study phases is illustrated in Figure 2.

### 3.1 Adherence

The overall study protocol adherence to timing of assessments for each participant is detailed in Table 4.

**TABLE 4:** Overall study protocol adherence to timing of assessment visits: Functional and clinical assessments, neurophys Ax1, and neurophys Ax2.

Participant	Functional and clinical Ax	Neurophys Ax1	Neurophys Ax2
P1	0.49	0.40	0.48
P2	0.63	0.65	0.65
P3	0.25	0.27	0.25
P4	0.54	0.54	0.54
P5	0.69	0.69	0.69
P6	0.59	0.64	0.63
P8	0.40	0.40	0.40
P9	0.26	0.26	0.25

**FIGURE 3:**
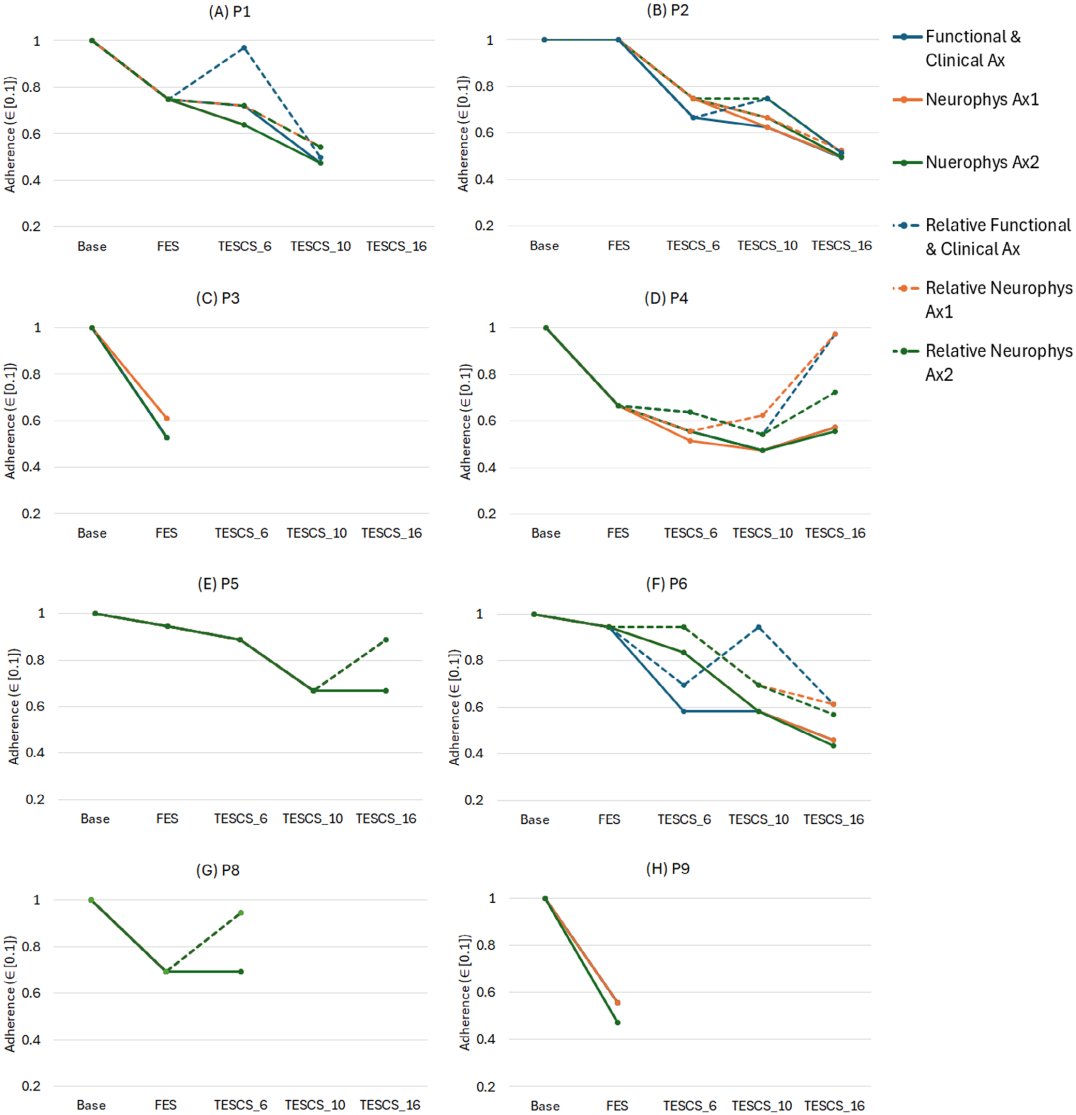
Study phase adherence and relative adherence for each participant (A–H) for assessment outcomes (neurophys Ax1, neurophys Ax2 and functional and clinical Ax). Study phase adherence (solid lines) accounts for knock on effects between a therapy block (phase) and the corresponding assessment timepoint for an outcome measure. Whereas, the relative phase adherence (dashed lines) does not account for knock on effects. [Color figure can be viewed at wileyonlinelibrary.com]

Participant study phase adherence and relative phase adherence are illustrated in Figure 3. Overall, adherence to the study protocol declined as the study progressed, with most participants showing a steady decrease in study phase adherence. The only exception was P4, who improved adherence during the final TESCS‐ABT phase.

Relative phase adherence provides a comparison between study phases (therapy blocks), offering insight into where adherence was highest and lowest for each participant.

Overall, adherence patterns varied across participants who completed the study (P2, P4, P5, and P6), though most showed a decline over time, except for P4, who improved in the final phase. For P2, adherence declined during TESCS‐ABT 1 but improved for functional and clinical assessments in TESCS‐ABT 2 before dropping again in the final phase (Figure 3B). P4 showed a steady decline from FES conditioning through TESCS‐ABT 1 and TESCS‐ABT 2 for neurophysiological Ax2 and functional and clinical Ax while adherence to neurophysiological Ax1 improved. Adherence to all assessments improved in the final phase for P4 (Figure 3D). P5 experienced a gradual decrease in adherence throughout the study, except for a slight improvement at the end to neurophysiological Ax2 (Figure 3E). For P6, relative adherence to functional and clinical assessments improved in TESCS‐ABT 2 before declining again, while neurophysiological assessment adherence remained stable early on but dropped in later phases (Figure 3F).

Relative adherence declined among participants who did not complete the study (P1, P3, P8, and P9). Improvements in relative adherence to functional and clinical Ax and neurophysiological Ax2 were shown in TESCS‐ABT 1 for P1 and P8, respectively (Figure 3A,G).

### 3.2 Maximum Voluntary Effort

#### 3.2.1 Inter‐Muscular Coherence

**FIGURE 4:**
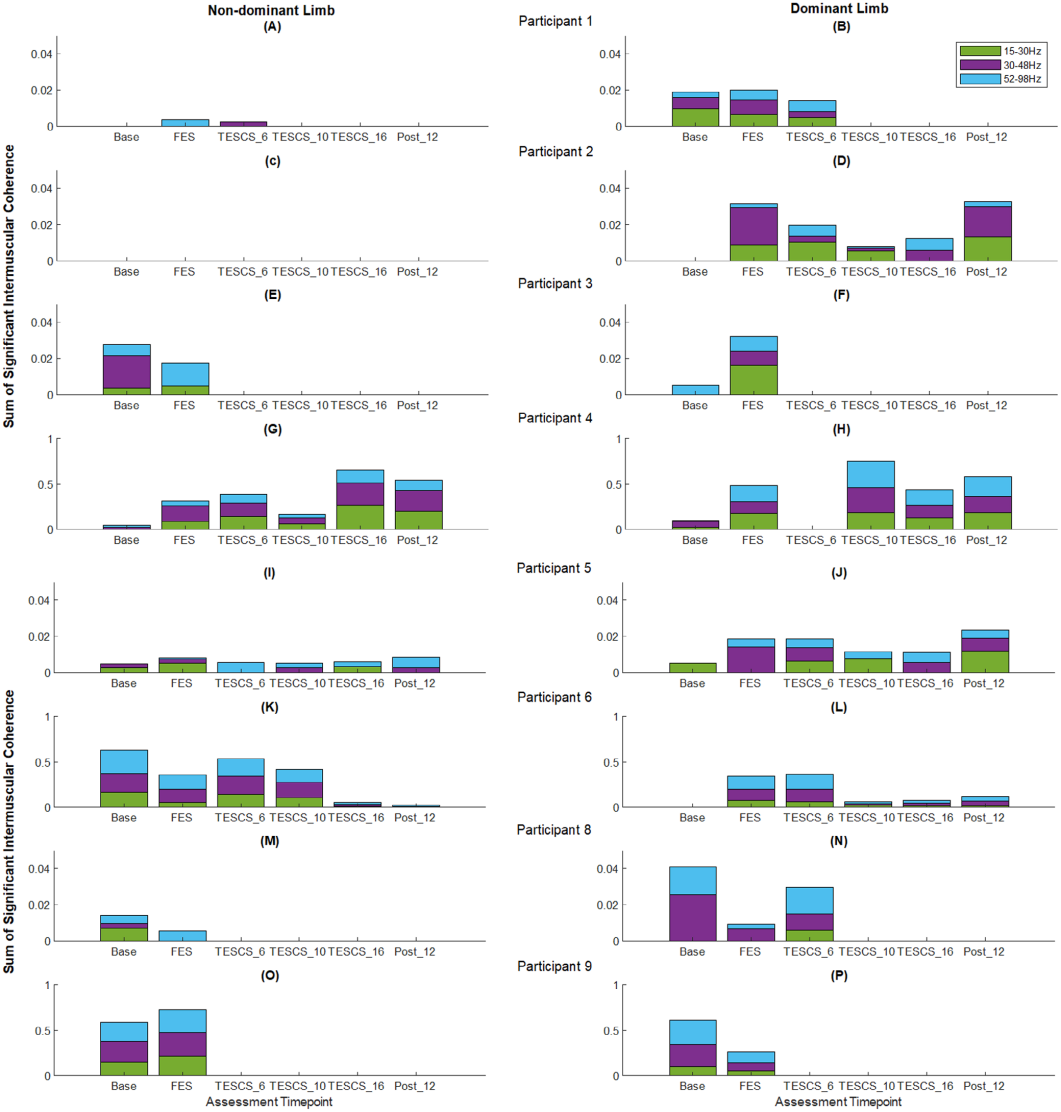
(A–P) Sum of significant (above 95% CI) beta‐, low gamma‐ and high gamma‐band inter‐muscular coherence (IMC_area_) of dominant (right column) and non‐dominant (left column) muscles of interest for each participant. [Color figure can be viewed at wileyonlinelibrary.com]

The sum of significant IMC in the beta‐, low gamma‐, and high gamma‐bands between muscles of interest for each participant across study timepoints is shown in Figure 4. Although participants exhibited significant IMC (above the 95% CI) across all frequency bands, beta‐ and low gamma‐band IMC showed greater variability across time points and participants.

Participants who completed the full study protocol exhibited improvements in high gamma‐band (52–98 Hz) IMC in the dominant limb following 6 weeks of TESCS‐ABT with further improvements after 16 weeks. P2 and P5 showed increases of 168% and 21% (Figure 4D,J), respectively, after 16 weeks TESCS‐ABT compared to post‐FES conditioning. P4 showed a 61% increase in high frequency IMC in the dominant limb, peaking at 10 weeks before returning to pre‐TESCS levels (Figure 4H). While P6 demonstrated a 17% increase after 6 weeks, this declined by 16 weeks TESCS‐ABT (Figure 4H). Only P4 and P5 retained improvements in the dominant limb at follow up (Figure 4H,J, respectively).

In the non‐dominant limb, two of four participants who completed the study showed increased (P4, 206%; P5, 249%) high gamma‐band IMC after 16 weeks TESCS‐ABT compared to pre‐TESCS (Figure 4G,I, respectively). P2 exhibited no detectable muscle activity in this limb throughout; therefore, he was excluded from analysis (Figure 4C). Again, P6 showed a 21% increase after 6 weeks in this limb, but this effect diminished by 16 weeks TESCS‐ABT (Figure 4K). As with the dominant limb, only P4 and P5 retained high gamma‐band IMC improvements in the non‐dominant limb at follow‐up (Figure 4G,I, respectively).

Improved high gamma‐band IMC was also observed for participants who did not complete the entire study but received TESCS‐ABT (P1 and P8). After 6 weeks of TESCS‐ABT, P1 showed an 8% increase in the dominant limb (Figure 4B), and P8 exhibited an increase in both the dominant (413%) and non‐dominant (28%) limbs compared to post‐FES conditioning (Figure 4M,N, respectively). Significant IMC in the non‐dominant limb of P1 was only observed following FES conditioning and disappeared after 6 weeks of TESCS‐ABT, with IMC shifting to the low gamma‐band (30–48 Hz), as shown in Figure 4A.

Two participants (P3 and P9) were only assessed at baseline and after FES conditioning. P3 showed increased high‐gamma IMC in both the dominant (51%) and non‐dominant (100%) limbs from baseline to after FES conditioning (Figure 4E,F, respectively). Whereas, P9 displayed opposing trends, with a 55% decrease in the dominant limb and a 21% increase in the non‐dominant limb (Figure 4O,P, respectively).

#### 3.2.2 MVErms

**FIGURE 5:**
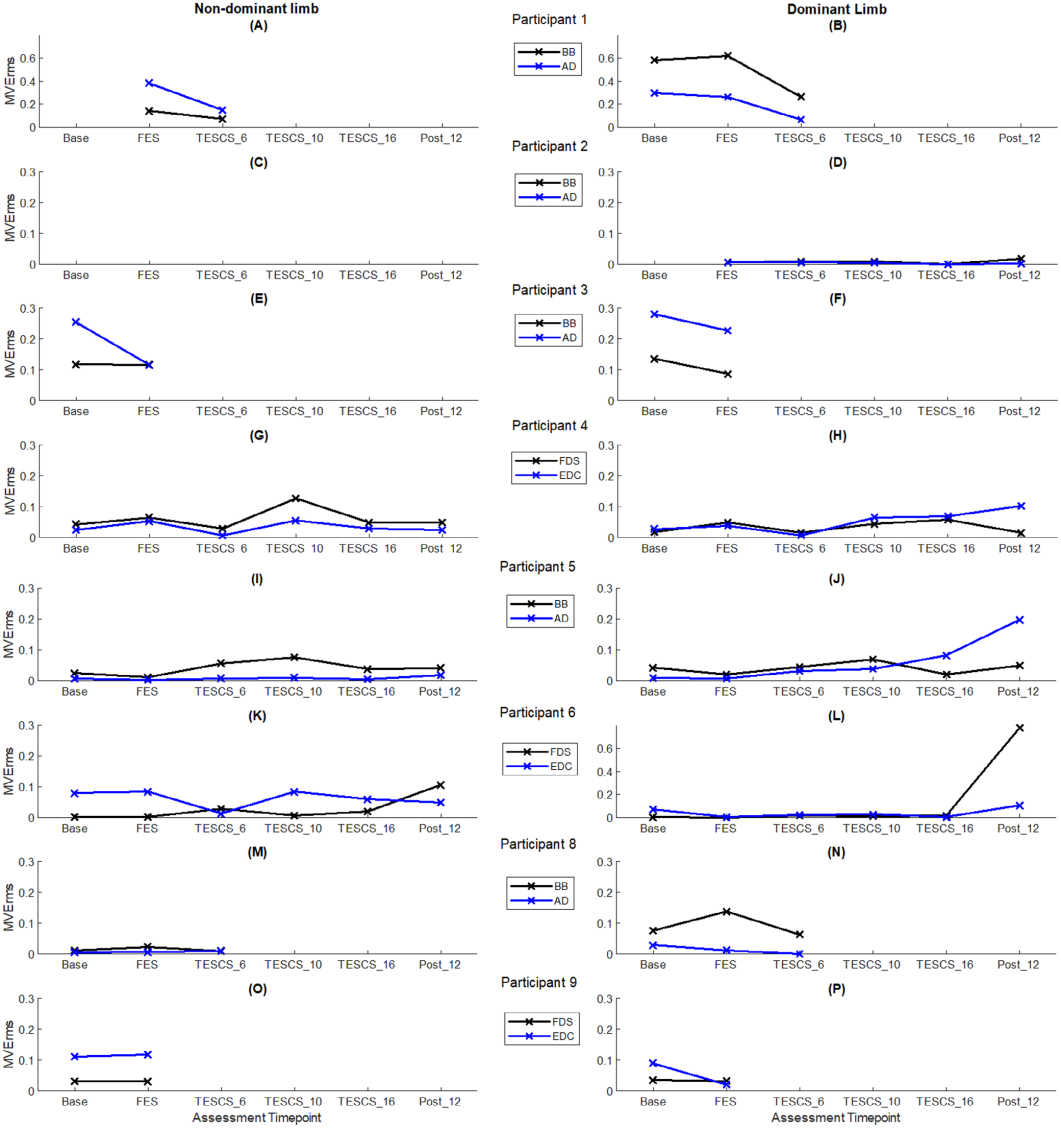
The maximum root mean square (rms) value of maximum voluntary effort trials using a dynamometer for each participant (A‐P). Dominant (right column) and non‐dominant (left column) muscles of interest for each participant are presented. AD, anterior deltoid; BB, biceps brachii; EDC, extensor digitorum communis; FDS, flexor digitorum superficialis. [Color figure can be viewed at wileyonlinelibrary.com]

Figure 5 illustrates the MVErms at each assessment timepoint for the dominant (right column) and non‐dominant (left column) muscles of interest for each participant.

Participants who completed the full study protocol demonstrated varying MVErms responses following TESCS‐ABT. In the dominant limb, a slight increase in MVErms was observed in both muscles of P4 (Figure 5H) after 16 weeks TESCS‐ABT, and the BB of P5 after 10 weeks, although this declined after 16 weeks but remained above pre‐TESCS levels (Figure 5J). The dominant muscles of P2 and P6 (Figure 5D,L, respectively) and the dominant AD of P5 (Figure 5J) showed little change throughout the study. Improvements in the dominant muscles were sustained at follow‐up for P4's EDC, P5's BB, and both muscles of P6.

In the non‐dominant limb, an improvement in MVErms was observed after 16 weeks of TESCS‐ABT in the AD for P5 and FDS for P6 compared to pre‐TESCS (Figure 5I,K, respectively). In contrast, both muscles of P4 showed a decrease after 16 weeks TESCS‐ABT, although the greatest MVErms were observed after 10 weeks for this participant (Figure 5G). Data from the non‐dominant muscles of P2 were excluded due to weak muscle contractions that could not be distinguished from noise (Figure 5C). Improvements in the non‐dominant muscles were sustained at follow up for both muscles of P5 and the FDS of P6.

Participants who did not complete the study but received TESCS‐ABT (P1 and P8) exhibited decreased MVErms in both the dominant and non‐dominant limbs after 6 weeks of TESCS‐ABT compared to pre‐TESCS (Figure 5A,B,M,N, respectively). Similarly, P3 and P9 (Figure 5E,F,O,P, respectively)remained unchanged or showed a decrease in MVErms from baseline to after FES conditioning.

### 3.3 Motor Evoked Potentials

#### 3.3.1 Cortical MEPs

**FIGURE 6:**
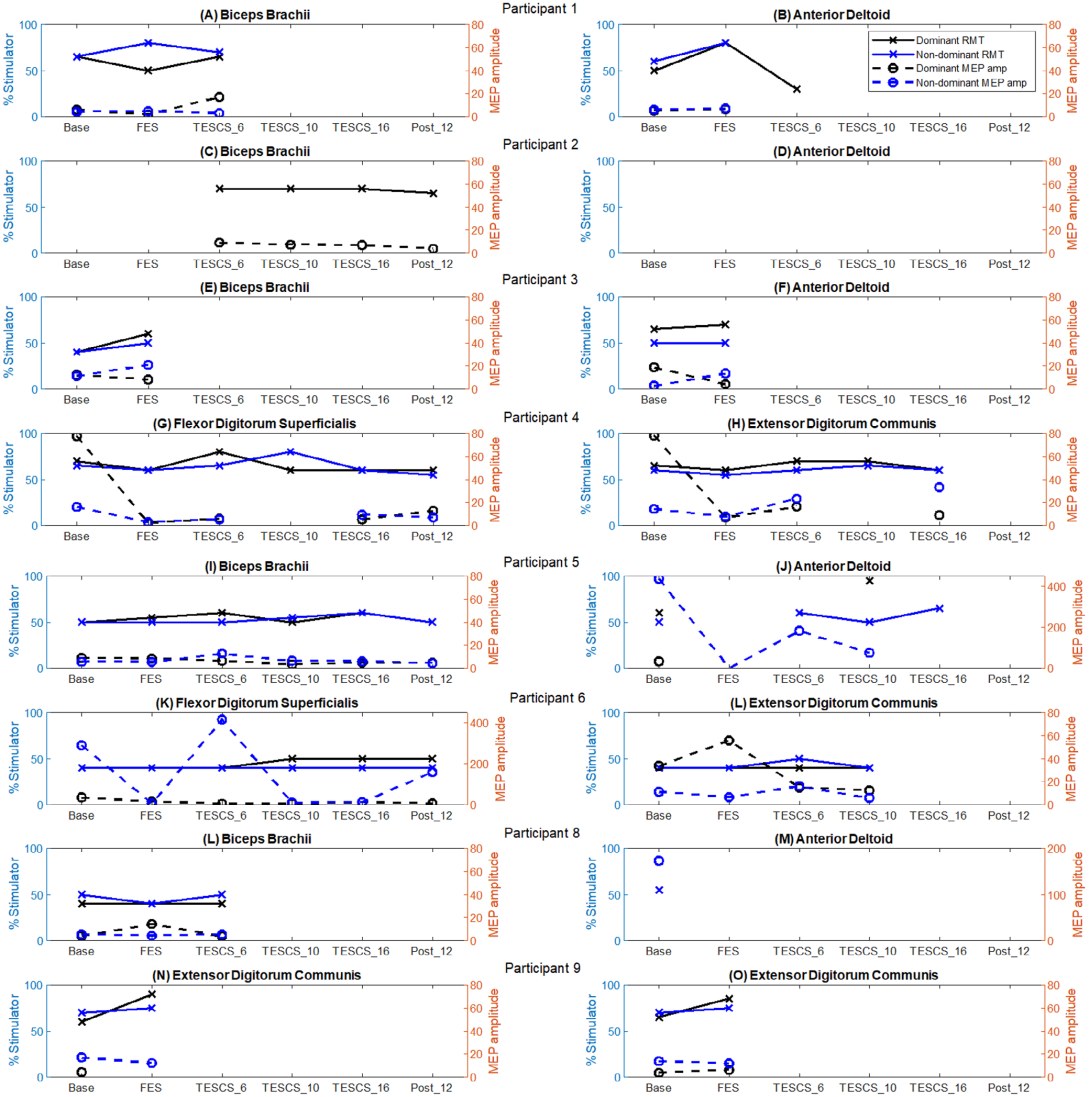
Resting motor threshold (RMT) and cortical MEP amplitude at 110% of RMT for the dominant (black lines) and non‐dominant (blue lines) muscles of interest of each participant [P1 (A, B), P2 (C, D), P3 (E, F), P4 (G, H), P5 (I, J), P6 (K, L), P8 (L, M), P9 (N, O)]. MVErms used for normalization of peak‐to‐peak amplitude. [Color figure can be viewed at wileyonlinelibrary.com]

Figure 6 shows the resting motor threshold (RMT) and normalized MEP amplitude at 110% RMT across study timepoints for each participant's muscles of interest.

No improvement was observed in the dominant limbs of participants who completed the entire study (P2, P4, P5, and P6), with MEP amplitude and RMT of dominant muscles fluctuating or remaining stable throughout. Unexpectedly, P4's dominant muscles had the highest MEP amplitude at baseline (Figure 6E,F). In contrast, these participants showed more improvement in the non‐dominant limb, with increased MEP amplitude observed after 16 weeks of TESCS‐ABT in the EDC for P4 and the FDS for P6 (Figure 6H,K, respectively) compared to the FES conditioning phase. A peak in MEP amplitude was seen in the BB of P5 (Figure 6I) and both muscles of P6 (Figure 6K,L) after 6 weeks of TESCS‐ABT. However, this decreased after 16 weeks, remaining above pre‐TESCS levels for the FDS of P6, but below pre‐TESCS levels for P5. The EDC of P6 also dropped below pre‐TESCS levels after 10 weeks. Improvements in MEP amplitude were retained at follow up for the FDS of P6 only.

Participants who received TESCS‐ABT but did not complete the study (P1 and P8) exhibited fluctuations in RMT and MEP amplitude. A decrease in RMT was observed in the dominant AD of P1 after 6 weeks of TESCS‐ABT compared to post‐FES conditioning (Figure 6B). An increase in MEP amplitude was observed in the dominant BB of P1 after 6 weeks of TESCS‐ABT compared to pre‐TESCS (Figure 6A). In contrast, the dominant BB of P8 showed a slight decrease in MEP amplitude after 6 weeks of TESCS‐ABT compared to pre‐TESCS, although it remained similar to the baseline value (Figure 6M).

The RMT of the BB in both limbs and the dominant AD of P3, as well as both muscles of the dominant and non‐dominant limbs of P9, increased from baseline to after FES conditioning (Figure 6E,F,O,P). MEP amplitude in P3 increased in the non‐dominant muscles but decreased in the dominant muscles from baseline to the end of FES conditioning (Figure 6E,F). For P9, the MEP amplitude slightly decreased in the non‐dominant FDS and EDC, while the dominant EDC showed a slight increase (Figure 6O,P).

A Spearman's correlation analysis showed no significant relationship between cortical MEP amplitude and high gamma‐band IMCarea (*ρ* = 0.18, *p* = 0.09). Additionally, no significant correlation was found between stimulation intensity at 110% RMT and MEP amplitude (*ρ* = −0.97, *p* = 0.51), indicating that increases in stimulation intensity due to higher RMTs did not systematically lead to higher MEP amplitudes.

#### 3.3.2 Spinal MEPs

**FIGURE 7:**
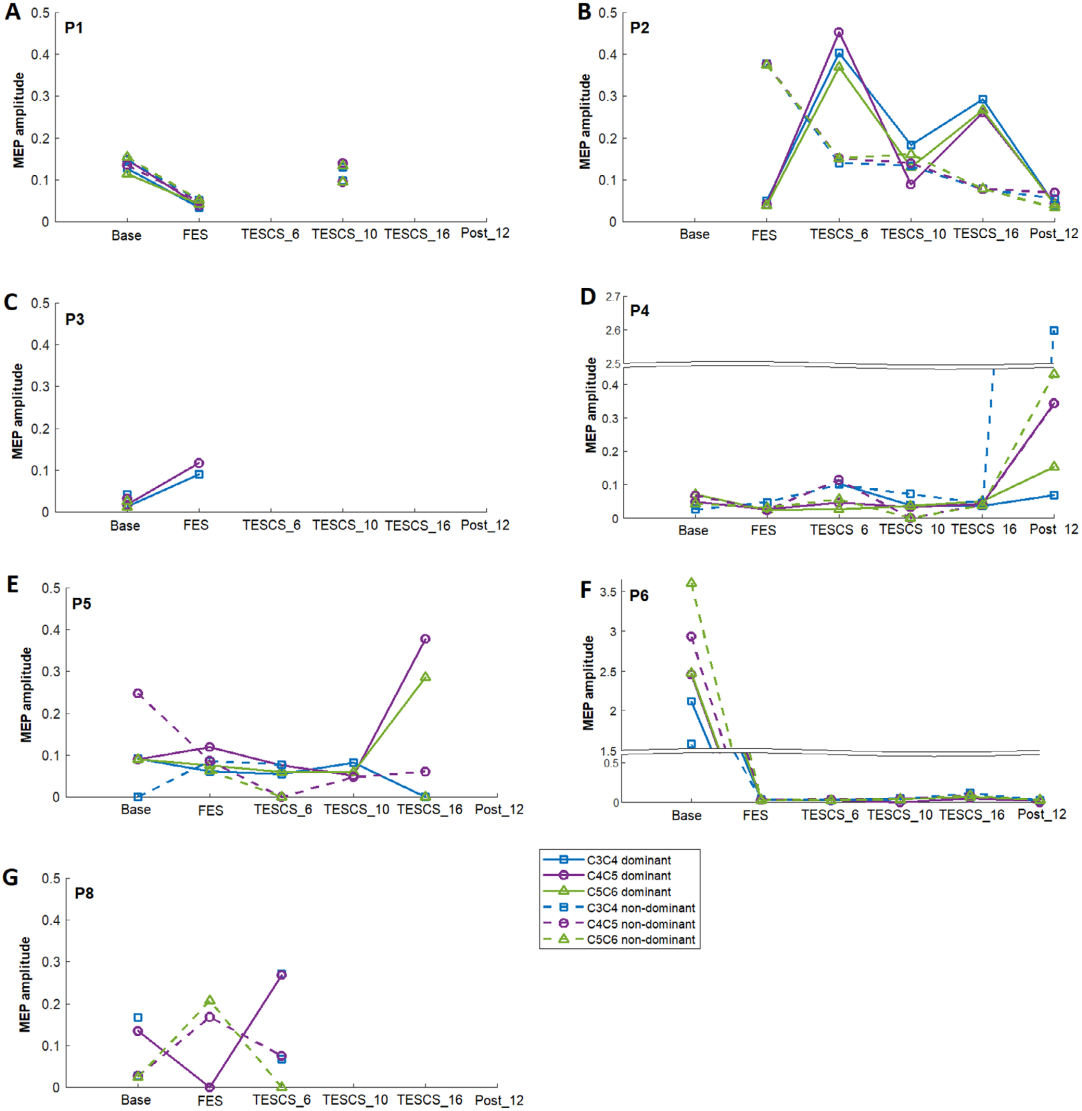
Spinal MEP amplitude at 110% of RMT for the dominant (solid lines) and non‐dominant (dashed lines) FDS muscle of each participant [P1 (A), P2 (B), P3 (C), P4 (D), P5 (E), P6 (F), P8 (G)]. Evoked potentials from supra‐maximal peripheral nerve stimulation of the median nerve at the elbow were used for normalization of peak‐to‐peak amplitude. [Color figure can be viewed at wileyonlinelibrary.com]

Figure 7 shows the normalized amplitude of FDS sMEPs at 110% RMT across study timepoints for each participant, excluding participant 9, who only showed a response at baseline in the left FDS when stimulating the C3–C4 spinal level. Only the spinal levels used as stimulation sites in TESCS‐ABT sessions are presented here, with full sMEP results available in Figure S3.

For participants who completed the entire study (P2, P4, P5, and P6), the amplitude of sMEP in the dominant limb was greater after 16 weeks TESCS‐ABT compared to pre‐TESCS for at least one spinal level (Figure 7B,D–F). Unexpectedly, the greatest sMEP amplitude was observed at follow‐up for P4 (Figure 7D) and at baseline for P6 (Figure 7F).

In the non‐dominant limb, less improvement in sMEP amplitude was seen across the participants. For P2, the sMEP amplitude decreased overall from pre‐TESCS to after 16 weeks of TESCS‐ABT across all spinal levels (Figure 7B). P4 showed fluctuations, with the greatest amplitude again at follow‐up (Figure 7D). For P5, a decrease was observed at the C4–C5 level, which was the only spinal level to elicit a consistent response throughout (Figure 7E). As with the dominant limb, P6 showed the greatest sMEP amplitude at baseline, with an overall increase from after FES conditioning to after 16 weeks of TESCS‐ABT (Figure 7F).

Results for participants who didn't complete the study were variable. For P1, sMEP amplitudes decreased from baseline to post‐FES conditioning across all spinal levels in both limbs, then returned to baseline levels after 10 weeks of TESCS‐ABT (Figure 7A). Due to time constraints, the spinal MEP assessment was not conducted after 6 weeks of TESCS‐ABT for this participant. For P3, a response was observed only in the dominant limb at both C3–C4 and C4–C5 after FES conditioning, with the C3–C4 response larger and both increased compared to baseline (Figure 7C). P8 showed an increase in sMEP amplitude at C4–C5 in the dominant limb after 6 weeks TESCS‐ABT compared to pre‐TESCS, while the non‐dominant limb showed a decrease at C4–C5 and C5–C6 (Figure 7G). At baseline, P9 showed a response only in the non‐dominant limb at C3–C4, with a normalized peak‐to‐peak amplitude of 0.0530, but no response was observed at any other site or timepoint.

A Spearman correlation analysis revealed no significant relationship (*ρ* = −0.11, *p* = 0.10) between stimulation intensity at 110% RMT and sMEP amplitude, suggesting that increases in stimulation intensity due to higher RMT values did not result in systematically larger sMEPs.

### 3.4 Somatosensory Evoked Potentials

Median and ulnar SSEP of both limbs were absent in all participants throughout the study. A representative example of SSEP recording from one participant can be found in Figure S4.

### 3.5 GRASSP Assessment

**FIGURE 8:**
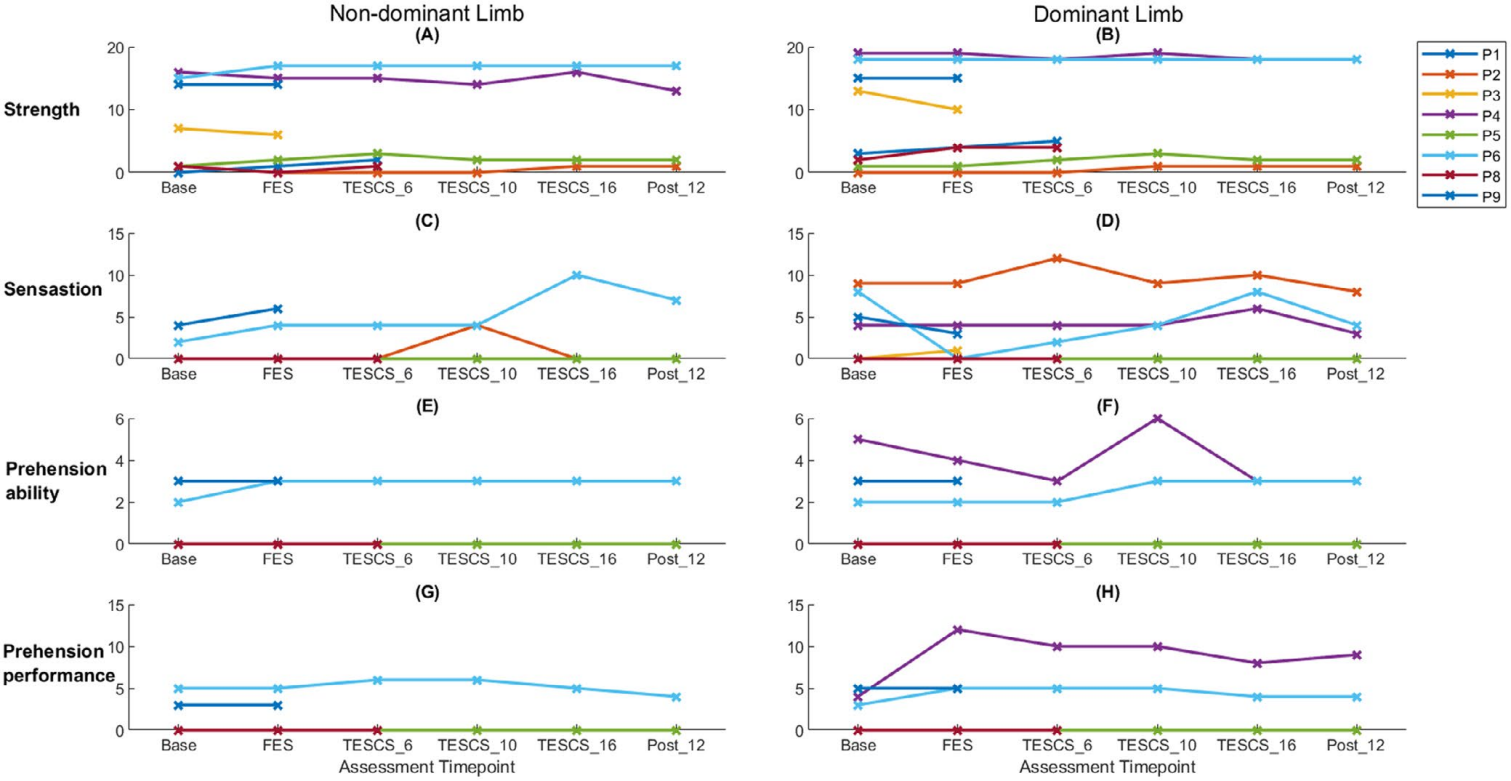
Participant GRASSP scores for strength (A, B), sensation (C, D), prehension ability (E, F) and prehension performance (G, H) for dominant (right column) and non‐dominant (left column) limbs. [Color figure can be viewed at wileyonlinelibrary.com]

There was little consistent improvement in GRASSP scores observed across participants, as illustrated in Figure 8A–H. However, all participants who completed the study showed improvement in at least one GRASSP subscale after 16 weeks of TESCS‐ABT compared to post FES conditioning. P2 exhibited a 1‐point improvement in strength and sensation of the dominant (left) limb (Figure 8A,C); A 2‐point increase in sensation of the dominant (right) limb and a 1‐point increase in strength of the non‐dominant limb was observed for P4 (Figure 8A,D respectively); P5 also improved strength of the dominant (right) limb by 1‐point (Figure 8B); and P6 showed a 6‐point improvement in sensation of the non‐dominant (right) limb (Figure 8D) and 1‐point improvement in prehension ability of the dominant (left) limb (Figure 8E). It should be noted that only the improvements in sensation in the dominant limb for P4 and P6 met the MCID, with only P6's improvement exceeding the MDD. Improvements were observed in at least one GRASSP subscale among participants who completed 6 weeks of TESCS‐ABT but did not finish the full study. P1 showed an increase of 2‐points, meeting the MCID but not the MDD, in strength after 6 weeks of TESCS‐ABT compared to baseline, with a 1‐point improvement relative to post‐FES conditioning in both the dominant and non‐dominant limbs (Figure 8A,B). P8 improved strength of the dominant limb by 1 point from baseline to after FES conditioning which was maintained after 6 weeks TESCS‐ABT (Figure 8B).

Additionally, the FES conditioning phase led to improvements even among participants who were not assessed after TESCS‐ABT or did not undergo TESCS‐ABT. P3 demonstrated a 1‐point increase in sensation in the dominant limb (Figure 8D), while P9 showed a 2‐point improvement in the non‐dominant (Figure 8C) limb after FES conditioning compared to baseline. Again, P9's improvement met the MCID but did not exceed the MDD.

## 4 Discussion

Our findings suggest that TESCS‐ABT may influence common neural drive to muscles, corticospinal and spinal excitability in individuals with chronic complete SCI, although the effects vary among participants and appear to be dependent on limb dominance. Specifically, TESCS‐ABT may increase high gamma‐band IMC and spinal excitability but produce an inhibitory effect at the cortical level in the presence of more extensive residual descending pathways. These results support the concept of “discomplete” spinal cord injuries where individuals clinically classified as complete retain subclinical residual connectivity at the level of injury [10, 11, 12]. While TESCS‐ABT appears to modulate motor pathways to varying degrees, it had no observed effect on the sensory pathway. This may reflect the distinct neurophysiological mechanisms that underlie motor and sensory recovery, indicating that while TESCS‐ABT may influence descending pathways in individuals with complete SCI, it does not appear to target or improve sensory pathways in this population. Nevertheless, the observed neurophysiological changes in this study, even in the absence of consistent functional improvements, highlight the potential of these residual, silent pathways to be leveraged through neuromodulatory therapies like TESCS‐ABT.

Participants demonstrated increases in high gamma IMC by 6 weeks of intervention, with further changes up to the 16‐week mark, indicating that both short‐ and longer‐term TESCS‐ABT may modulate IMC. Extensive evidence supports that beta‐ and gamma‐band IMC are of cortical origin and reflect common neural drive to muscles during coordinated motor tasks [18, 19, 50]. Thus, our results suggest that TESCS‐ABT may positively influence neuromuscular connectivity. As expected, baseline IMC in the high gamma band was greater in those with residual function; however, improvements were not consistently aligned with baseline neuromuscular state. Moreover, IMC gains did not correspond to higher stimulation intensities. Instead, adherence appeared more influential, with the greatest IMC gains after 16 weeks TESCS‐ABT corresponding to the highest overall study adherence (P5) and the second highest aligning with peak adherence during TESCS‐ABT 3 (P4). Both participants retained IMC improvements at follow‐up, suggesting engagement may impact neuromuscular adaptation and its sustainability. However, adherence alone cannot account for the lack of a consistent trend across timepoints and frequencies among participants, suggesting that responses to TESCS‐ABT vary across participants and may reflect individualized patterns of neuromuscular adaptation.

While increased gamma‐band IMC has been associated with improved locomotion in iSCI, indicating a role for enhanced corticospinal drive in motor function, this relationship was not observed in our complete SCI cohort [19, 50]. This may be due to the lack of intact descending motor pathways in complete SCI, which could limit the potential for IMC‐mediated neuromuscular coordination. Pizzamiglio et al. proposed that IMC in the 40–100 Hz frequency range reflects a neural strategy required for muscle coordination during motor adaptation [51]. Given that SCI disrupts neural pathways essential for effective motor coordination, compensatory mechanisms may be required to restore force production [16, 17, 18]. The observed high frequency IMC increases, independent of baseline neuromuscular state, suggest that this frequency band may reflect these compensatory processes rather than directly enhancing corticospinal drive in this SCI population. Thus, the relevance of IMC in complete SCI may be more complex than in iSCI, highlighting the need for further research to fully understand its role on motor control.

Although this is the first study to examine the effect of TESCS on IMC in SCI, prior research has linked gamma‐band IMC and corticospinal excitability in this population. Specifically, studies in iSCI have attributed the positive correlation between cMEP amplitude and gamma‐band IMC during walking to increased corticospinal drive and preserved corticospinal tract function [19, 50]. Although the results of this study do not directly support these findings, as no significant relationship was found between cMEP amplitude and high gamma‐band IMC, peak IMC coincided with peak cMEP amplitude in the non‐dominant limb of participants with lower injury levels (P4 and P6). This contrasts with expectations that the dominant limb would possess greater potential for improvements since it typically retains greater motor function, suggesting more extensive residual connectivity of descending pathways [52]. Since previous studies have not distinguished between dominant and non‐dominant limbs [19, 50], the implications of this finding remain unclear. Therefore, further research is required to determine the effect of limb dominance on corticospinal drive in the SCI population.

TESCS‐ABT did not enhance corticospinal excitability in the dominant limb, however it did increase spinal excitability. This aligns with findings from Benavides et al., of concurrent excitatory effects of TESCS at the spinal level and inhibitory effects at the cortical level in the dominant limb of individuals with iSCI. Despite cortical inhibition, they observed improved motor function, attributing it to TESCS influencing neuronal networks controlling antagonistic muscles, thereby enhancing performance [23]. Consistent with this, our study found improvements in at least one GRASSP subscore in the dominant limb after 16 weeks TESCS‐ABT compared to before. However, in contrast with the study by Benavides et al., GRASSP improvements in our study only met the MDD in one participant (P6), and neurophysiological and functional gains did not translate into meaningful functional recovery. Therefore, it could be that limited supraspinal connectivity in complete SCI may be insufficient for neuromodulatory effects to translate into functional improvements. Brain–computer interface priming before TESCS has been used to target supraspinal networks in chronic complete SCI, demonstrating improved functional outcomes; however, its effect on corticospinal excitability was not measured [30]. Perhaps priming cortical networks is required in this population to encourage supraspinal connectivity prior to TESCS‐ABT. Future work should consider exploring alternative approaches, such as priming, to enhance neuromodulatory effects of TESCS and functional recovery in complete SCI.

Similarly, a study by Zhang et al. reported increased spinal excitability in the dominant limb of an individual with AIS A after 18 sessions of TESCS over 8 weeks. However, unlike our findings, this heightened excitability translated to bilateral functional improvements across all GRASSP sub tests and hand grip strength [7]. The disparity in results may stem from differences in electrode configuration and stimulation parameters. The monophasic current used by Zhang et al. may provide the benefit of being able to facilitate stronger neuromodulatory effects due to the cumulative depolarisation of spinal neuronal networks. However, it has been suggested that the initial therapeutic approach for TESCS should prioritize biphasic stimulation, as it may help prevent the development of excessive muscle tone that could impede motor function and has been shown to facilitate voluntary movement before triggering unwanted muscle twitches [53]. Since this waveform reduces the risk of burns at the electrode site and is generally considered smoother and less painful than monophasic stimulation, it was deemed more appropriate for our study. Furthermore, Zhang et al. applied stimulation above and below the level of injury using two round cathode electrodes, whereas our study employed more targeted stimulation with a single cathode electrode. Although previous research has shown one electrode to be effective for neuromodulation [23, 54], this clinical population may require broader spinal cord activation to facilitate motor signal transmission beyond the level of injury. Therefore, future research should explore the effects of both multi‐site and single‐site stimulation in individuals with AIS A SCI.

Although our study found some transient improvement in muscle activation, as measured by MVErms, and GRASSP outcomes, there was no clear trend and gains did not exceed the MDD or translate into meaningful functional improvements. This contrasts with Moritz et al., who reported TESCS rehabilitation therapy resulted in improvements, exceeding the MCID, in strength and functional outcomes in individuals with iSCI [6]. Both studies followed similar protocols, including a conditioning period prior to commencing TESCS‐assisted rehabilitation sessions. Similar to our study, each 60‐min TESCS session included specialist‐selected exercises tailored to the participant's ability and progressively adjusted as they improved, with active assistance when voluntary movement was minimal or absent. Since protocol differences are unlikely to explain the disparity in results, patient demographics may have influenced the results. Moritz et al. included iSCI participants with moderate strength and prehension (GRASSP‐strength score of ≥ 30 and a GRASSP‐prehension score of ≥ 10), whereas our study involved individuals with chronic, motor‐complete SCI, who had severely limited or no voluntary movement. This likely restricted exercise progression and intensity in our cohort, while participants in the Moritz et al. study may have engaged in more complex and higher‐intensity movements over time. Additionally, our longer intervention period may have contributed to lower adherence, potentially influencing results. Nonetheless, the lack of consistent outcomes across assessment timepoints suggests that adherence alone is unlikely to explain these findings. Since individuals with complete SCI have limited residual connections extending beyond the level of injury compared to those with iSCI, it could be that the extent of these spared supraspinal connections are important for neurophysiological changes induced by TESCS to translate into meaningful functional improvements. Balaguer et al., emphasized that subthreshold sensory inputs from TESCS generate motor neuron action potentials only in the presence of supraspinal drive [14]. Thus, individuals with less severe SCI and greater residual function may have a higher capacity for meaningful recovery with TESCS‐ABT. Identifying the optimal target population should be a priority for future research.

Given that sensory activation is a key mechanism underlying the neuromodulatory effects of TESCS, the absence of SSEP responses in all participants may have contributed to the limited and variable effects observed in our study. Preserved ascending sensory connectivity enables sensory feedback that supports synaptic reorganization and plasticity, ultimately enhancing voluntary motor function. In complete SCI, the lack of ascending sensory information, as indicated by absent SSEPs, disrupts this feedback loop, impairing plasticity and limiting the efficacy of neuromodulation. This may help explain the disparity between the results of our study in complete SCI and previous findings in individuals with incomplete SCI. Nevertheless, it has been suggested that TESCS can still induce neuromodulatory effects even with low levels of sensory input when paired with motor tasks, as repetitive sensory‐motor learning can drive the reorganization of spinal circuits [55]. This mechanism may help account for reports of motor improvements following TESCS in an individual with chronic complete SCI [7]. However, in the absence of supporting neurophysiological data, it remains unclear whether the same underlying processes are responsible. Therefore, it is possible that individuals with incomplete SCI, or those with more residual intact pathways, may be likely to benefit from TESCS‐ABT therapy due to their greater potential for sensory feedback‐driven plasticity.

The health benefits of FES cycling, including improvements in muscle mass and cardiopulmonary function, are well established and it is widely used in clinical SCI rehabilitation. Beyond these health benefits, FES cycling also facilitates neuroplastic recovery through stimulation of mixed peripheral nerves, activating efferent fibers to produce muscle contractions and afferent fibers to deliver sensory feedback that promotes neural plasticity. This mechanism is supported by previous studies demonstrating that prolonged upper and lower limb FES cycling can lead to significant motor function improvements in individuals with chronic complete and incomplete SCI [56, 57]. Notably, some participants in our study demonstrated improvements in both neurophysiological and functional outcomes after 6 weeks of FES cycling, suggesting that this phase achieved its intended conditioning effect. However, it is also possible that some participants reached a plateau in neuromodulatory responsiveness during this phase, which may have contributed to the variability in responses observed during the subsequent TESCS‐ABT phase. A similar observation was reported by Tefertiller et al., who implemented FES prior to TESCS and suggested that this design may have led to a ceiling effect, contributing to the variability in upper limb recovery among individuals with AIS B–D SCI [58]. This is further supported by the previously mentioned study, which observed motor improvements following TESCS in an individual with chronic complete SCI, where no FES‐only phase preceded the intervention [7]. Therefore, it is possible that omitting this preparatory stage may help mitigate potential ceiling effects and enhance responsiveness to TESCS in this clinical population.

Given that FES directly stimulates peripheral nerves, it may be a more suitable neuromodulatory strategy for individuals with complete SCI, in whom the integrity of ascending and descending spinal pathways is severely compromised. However, the non‐physiological recruitment of muscle fibers during FES leads to rapid fatigue, limiting its effectiveness for prolonged use [59]. Thus, combining FES with TESCS may offer synergistic benefits by engaging both peripheral and central neural circuits, thereby enhancing neuroplasticity and functional recovery. This multimodal approach may be particularly advantageous in individuals with complete SCI, for whom TESCS alone may have limited efficacy. Recently emerging research on this combined strategy suggests its potential to enhance motor function [60, 61]. However, its effectiveness in individuals with SCI has yet to be established, highlighting the need for further research.

Overall, the inconsistency in neurophysiological changes and the limited functional translation suggests that individuals with chronic, complete SCI may not experience the same benefits from TESCS‐ABT as those with greater residual function. Furthermore, limb dominance seems to influence the response to TESCS‐ABT, likely due to more extensive residual supraspinal connectivity, which may impact neuromodulatory effects. Future research should explore individualized factors influencing neuromodulatory responsiveness to TESCS‐ABT to better identify candidates who may benefit from this intervention.

### 4.1 Limitations

A limitation of this study is the small sample size, a larger number of participants would be necessary to consolidate results. Additionally, conducting a large study with this patient population presents significant challenges due to their complex medical needs, which may result in missed sessions and difficulty maintaining consistent participation. The severity of their injuries requires extensive care, including assistance with daily activities and transport, further complicating logistics. These factors coupled with the time commitment required from the participants makes adherence to the study protocol difficult which may influence results. Given the complexity of neuroplastic changes, particularly in AIS A individuals, a prolonged intervention was chosen to maximize the likelihood of serving meaningful adaptations. However, the feasibility of such a long‐duration study in this SCI population was limited, as it resulted in a high dropout rate, with only four participants completing the study, and poor adherence. Future studies should take this into consideration when designing study protocols for individuals with complete SCI.

While it is common practice to set stimulation intensity based on participant tolerance [9, 30, 62, 63, 64], a limitation of this approach is that some individuals may not reach an intensity sufficient to optimally activate spinal neuronal networks. In this study, we confirmed that TESCS remained below motor threshold by the absence of visible muscle contractions; however, low intensities were not specifically controlled, which may have influenced the extent of dorsal root activation. Consequently, the stimulation intensity may not have been strong enough to elicit a measurable effect in some participants. Future studies would benefit from determining intensity relative to motor threshold and confirm dorsal root activation using neurophysiological testing, ensuring a more systematic approach to setting stimulation parameters and allowing objective comparisons across studies.

## 5 Conclusion

In conclusion, the inconsistency in results highlights the complexity of the neurophysiological effects of TESCS‐ABT in chronic complete SCI. In contrast to studies of iSCI, neurophysiological changes did not translate into meaningful functional improvements, suggesting that individuals with complete injuries may not experience the same degree of benefit as those with incomplete injuries. The variability across participants indicates that TESCS‐ABT may have limited efficacy for this SCI population, particularly in the absence of residual motor function. These findings highlight the need for future research to explore alternative approaches for this population and further investigate the neurophysiological effects and mechanisms of TESCS, particularly in individuals with iSCI, who may be more responsive to its neuromodulatory effects.

## Author Contributions

Conceptualisation: A.V. and M.P. Methodology: B.O., A.V., E.J.M., M.P., and E.L.M. Investigation: B.O., E.L.M., C.L., and L.C. Data analysis: E.L.M. and B.O. Results interpretation: E.L.M., A.V., E.J.M., and M.P. Drafted manuscript: E.L.M. Edited and revised manuscript: A.V., B.O., M.P., E.J.M., C.L., and L.C. Figure preparation: E.L.M.

## Conflicts of Interest

The authors declare no conflicts of interest.

## Supporting information


Data S1.
