# Supplementary material for: Neurophysiological Effect of Transcutaneous Electrical Spinal Cord Stimulation in Chronic Complete Spinal Cord Injury
Source: Artif Organs. 2025 Jun 30;49(12):1765–86. doi: 10.1111/aor.15050 (PMC12902743; doi:10.1111/aor.15050)
Supplement: Supplementary file 1 — Data S1. [file AOR-49-1765-s001.pdf]

Table S1 Predefined exercises for TESCS-ABT sessions. Exercises were selected at the discretion of a SCI specialist occupational therapist or physiotherapist, based on the participant's upper limb function.

Category	Activities
**Passive stretching**	
**Active and Active Assist Range of Motion**	- Active Range of Motion exercises with/without resistance (theraband, weights, manual)
**Gross Upper Limb Movement**	- Table top dusting (flat/inclined)<br>- Page turning<br>- Touch/reach to target<br>- Batting a balloon<br>- Throw/catch ball<br>- Theraband exercises<br>- Rolling theraputty<br>- Rolling ball (+/- incline)<br>- Lifting cones on/off shelf<br>- Puzzle tree<br>- Curtain Pole<br>- Number board<br>- Others – Bespoke activities
**Development of Wrist Flex/Ext and Pronation/Supination**	- Flex/extend to target<br>- Velcro roller<br>- Wringing putty/towel<br>- Pulling putty<br>- Flex/ext holding ball<br>- Solitaire<br>- Pronation/supination to target<br>- Others – Bespoke activities
**Development of Grasp**	- Grasp and release towel roll<br>- Acquire/release of cup<br>- Crumpling a napkin/towel<br>- Cane hoops<br>- Picking up and putting down cone<br>- Stacking cones<br>- Theraputty exercises<br>- Geometric stacker<br>- Pouring water from a cup<br>- Velcro roller<br>- Ergonomic hand exerciser<br>- Opening jars<br>- Towers of Hanoi<br>- Others – Bespoke activities
**Development of Pinch**	- Solitaire (varying sizes)<br>- Theraputty exercises<br>- Stacking with pinch (Jenga, Structuro, coloured pegs)<br>- Nuts and bolts<br>- Putting small objects in a jar

	- Threading buttons/lace up card<br>- Tearing paper<br>- Paperclips<br>- Tweezers<br>- Hamma beads<br>- Others – Bespoke activities
**Development of Finger Movements**	- Finger tapping<br>- Theraputty exercises<br>- Touch to target<br>- Typing on keyboard<br>- Piano<br>- Others – Bespoke activities
**Graded Function Focused Tasks**	- Feeding: (+/- feeding strap/modified cutlery) holding cutlery, picking up cutlery, stabbing target with fork, fork to mouth, cutting with knife<br>- Drinking: (+/- drinking cup/OB) acquire/release cylindrical grip, lift cup on/off table, cup to mouth, increase weight of cup, hydrant straw to mouth<br>- Use of mobile phone/tablet<br>- Putting glove on/off<br>- Grooming tasks: (+/- equipment) holding hair brush, brushing hair, holding toothbrush, applying toothpaste, brushing teeth<br>- Others – Bespoke activities

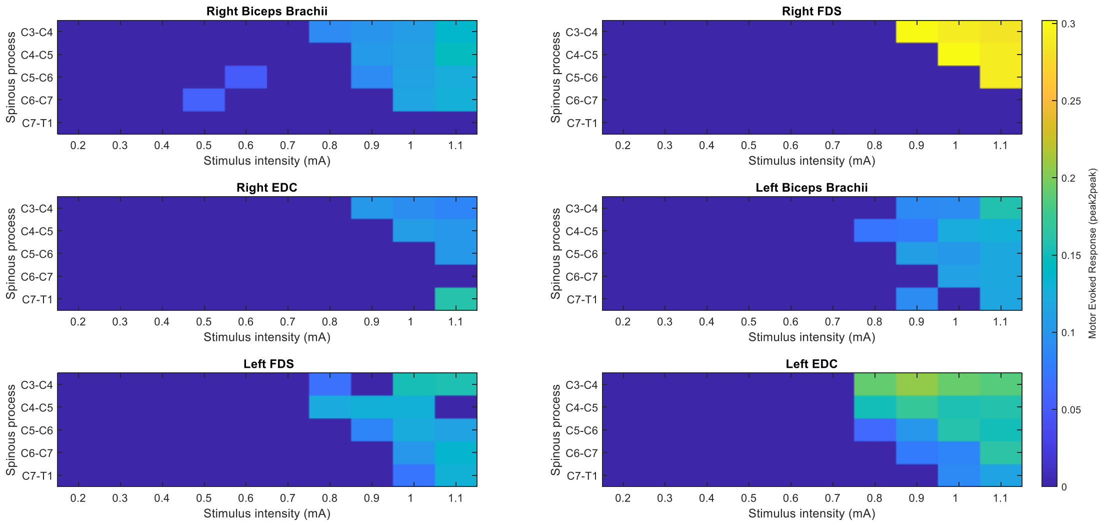

Figure S1 A representative example of a spinal map used to guide cathode electrode placement for TESCS-ABT sessions, helping to identify the optimal location based on the most efficient activation of target muscles during each activity. Spinal map of P2 after FES-conditioning phase.

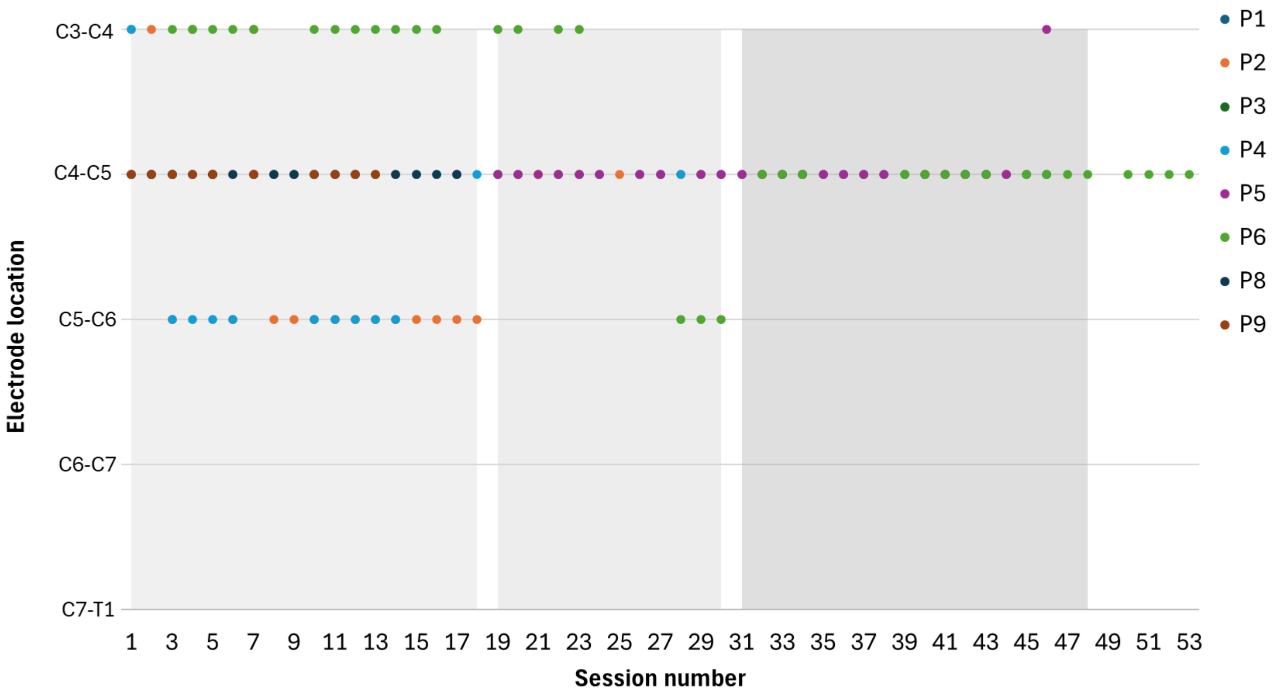

Figure S2 Participant cathode electrode position for each TESCS-ABT session.

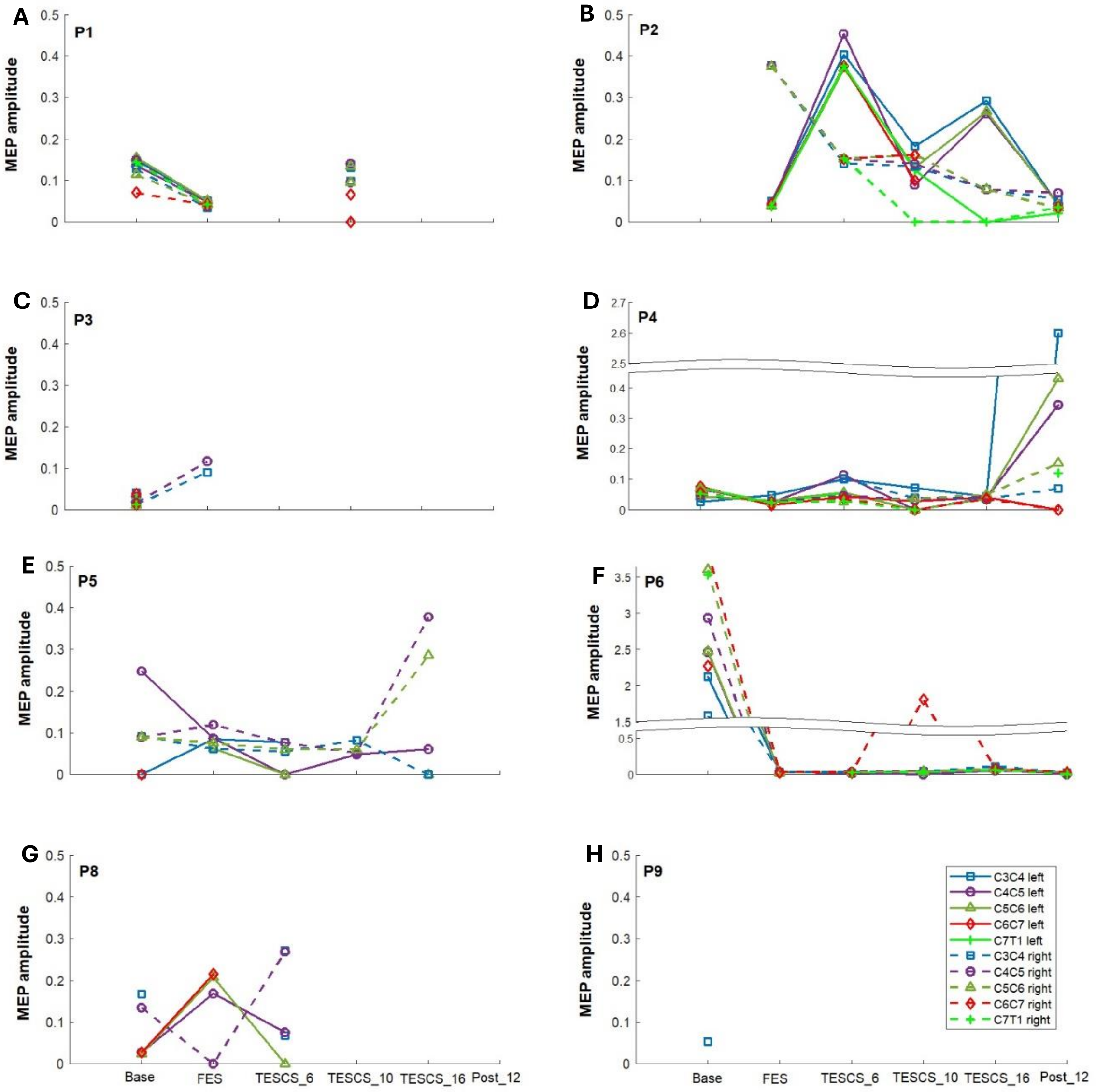

Figure S3 Spinal MEP amplitude at 110% of RMT for left and right FDS muscle of each participant (P1:A, P2: B, P3:C, P4: D, P5: E, P6: F, P8: G, P9:H) at assessment timepoints throughout the study. Evoked potentials from supra-maximal peripheral nerve stimulation of the median nerve at the elbow used for normalisation of peak-to peak amplitude.

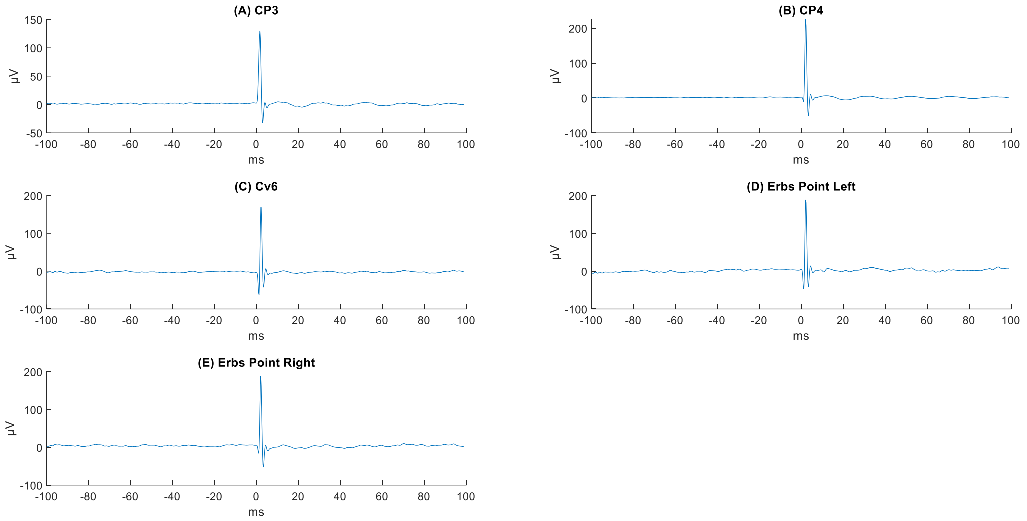

Figure S4 A representative example of median nerve SSEP recordings from (A) CP3 and (B) CP4, based on the international 10-20 EEG electrode positioning system, as well as from (C) the C6 spinal cord and (D) left and (E) right Erb's points. Shown is the SSEP of the left median nerve from Participant 6 (P6) after the FES-conditioning phase.

Table S2 Participant RMT stimulation intensity (mA) elicited by single pulse stimulation at C3-C4 spinal level.

Participant	Assessment timepoint					
	Base	FES	TESCS_6	TESCS_10	TESCS_16	POST_12
P1	25	27.6				
P2	3.5	30	27	28	29	32
P3	31	25				
P4	55	40	43	42	40	40
P5	34	40	34	33	37	38
P6	30	34	27	27	28	29
P8	34	30	37			
P9	35	29				
